# Collective events and individual affect shape autobiographical memory

**DOI:** 10.1073/pnas.2221919120

**Published:** 2023-07-11

**Authors:** Nina Rouhani, Damian Stanley, COVID-Dynamic Team, Ralph Adolphs

**Affiliations:** ^a^Division of Biology and Biological Engineering, California Institute of Technology, Pasadena, CA 91125; ^b^Division of Humanities and Social Sciences, California Institute of Technology, Pasadena, CA 91125; ^c^Derner School of Psychology, Adelphi University, New York, NY 11530

**Keywords:** collective memory, autobiographical memory, temporal memory, emotion, surprise

## Abstract

How do collective events shape how we remember our lives? We leveraged advances in natural language processing as well as a rich, longitudinal assessment of 1,000 Americans throughout 2020 to examine how memory is influenced by two prominent factors: surprise and emotion. Autobiographical memory for 2020 displayed a unique signature: There was a substantial bump in March, aligning with pandemic onset and lockdowns, consistent across three memory collections 1 y apart. We further investigated how emotion, using both immediate and retrieved measures, predicted the amount and content of autobiographical memory: Negative affect increased recall across all measures, whereas its more clinical indices, depression and posttraumatic stress disorder, selectively increased nonepisodic recall. Finally, in a separate cohort, we found pandemic news to be better remembered, surprising, and negative, while lockdowns compressed remembered time. Our work connects laboratory findings to the real world and delineates the effects of acute versus clinical signatures of negative emotion on memory.

## Significance

Global events can inflect our life trajectories, tuning memory for experiences that will not only define historical narrative but our personal life stories as well. Here, we characterized the dynamic between autobiographical and collective memory through a large-scale, longitudinal examination of how experienced and remembered emotion across 2020 influenced the content and structure of long-term memory. We found the pandemic onset to increase memory for that period while lockdowns compressed remembered time. Experienced emotion further amplified the shape and substance of memory; negative emotion, in particular, enhanced recall, while its more chronic markers, depression and posttraumatic stress disorder, predicted more blunted recall. Our results characterize how real-world surprise and emotion, experienced collectively and individually, shape memory, with implications for psychopathology.

Collective memories can sculpt a narrative of our past by filtering through the mundane to highlight the twists and turns that color and shape our personal autobiographies. Unexpected events, particularly those that are emotionally salient, hold special reins in memory by updating previous expectations and inciting new behavior. In neurobiological models of learning and memory, surprise, or “prediction error,” quantified by the extent to which outcomes deviate from expectations, is a powerful modulator of neural and cognitive mechanisms, boosting learning and memory across rewarding and aversive domains (1–7). High prediction errors can further split preceding and subsequent events into separate chunks (8–11), giving rise to distinct epochs in memory. When these unexpected turns are unpleasant or stressful, this mechanism can be adaptive by encouraging avoidance of similar events; however, it can also be debilitating, as in posttraumatic stress disorder (PTSD), where individuals are crippled by involuntary or intrusive thoughts stemming from experienced trauma (12).

Highly unexpected or stressful events can influence the collective memory of entire communities. “Flashbulb memories” for historically significant events (13), such as the assassination of Martin Luther King or 9/11, are marked by their vividness and confidence over time (while subject to distortion; 14–16). Nevertheless, whether collective events exert a profound influence on autobiographical memory depends on their impact in shifting individual life trajectories (“Transition Theory”: 17, 18), during which enhanced memory for specific events can benefit survival (19). Within this framework, the COVID-19 pandemic meets the criteria for an unexpected and high-impact collective event, further predicted by Transition Theory (18) to generate a bump in autobiographical memory. Studies of memories for such disasters are by their nature rare, likely to involve limited and geographically restricted sampling of participants, and often do not combine memory collection with other psychological measures over an extended period of time.

**Fig. 1.**
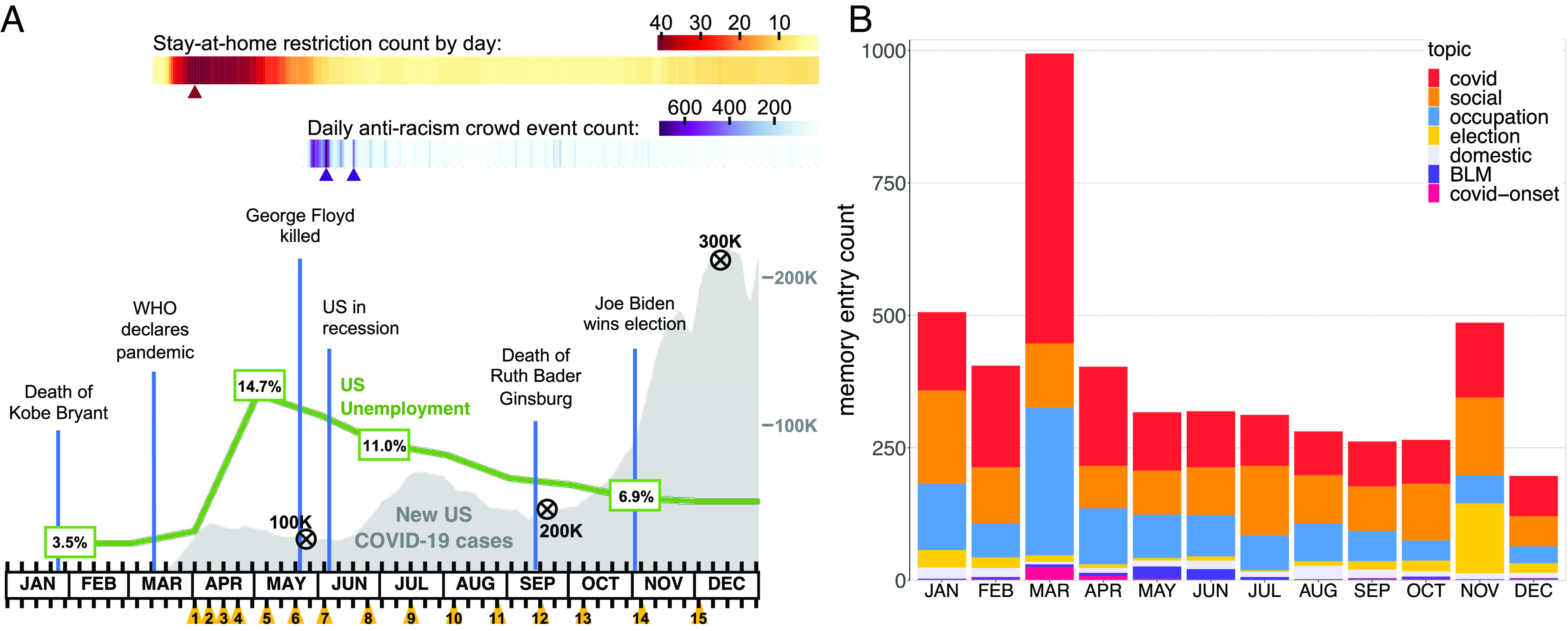
(*A*) Timeline of real-world events in the United States and COVID-Dynamic administrations across 2020. Orange triangles denote each wave administration (black tick marks depict weekly intervals). The gray curve indicates the daily 7-d average of new, confirmed COVID-19 cases in the United States, black encircled X’s on top of the curve indicate death milestones. The green line shows the monthly unemployment rate. The upper gradient (yellow-red) indicates the daily count of states with active stay-at-home restrictions (peak = 41). The lower gradient (blue-purple) shows the daily count of US antiracism crowd events initiated after the murder of George Floyd. Triangles below the gradients indicate local maxima for the various measures. Events of interest are indicated with vertical blue lines (credit: COVID-Dynamic Study, data are publicly available, see *SI Appendix*, section 5.1 for resources; 20). (*B*) Topic modeling for 2020 autobiographical memory distributed by month (memory recalled in December 2020, see *SI Appendix*, section 2.3 for topic modeling of all sets). Memory entry count (*Y* axis) represents the number of memory entries, grouped by topic category. Topics included categories related to personal matters (“social,” “occupation,” and “domestic”) as well as real-world events (“covid,” 2020 presidential “election,” Black Lives Matter movement (“BLM”), and “‘covid-onset” which describes the conditions of the early pandemic (e.g., toilet paper shortage); see *SI Appendix*, Table S2 for keywords associated with each topic. A similar pattern of topics was observed for all 2020 memory collections (but not for 2021 memory, which included different real-world topics, *SI Appendix*, Fig. S2).

Here, we collected a battery of measures characterizing the immediate and longer-term emotional states of individuals throughout 2020 as well as their subsequent memories for specific, dated events across 3 y. Subjects were recruited from a unique study that followed over 1,000 broadly sampled American adults, collecting a rich set of questionnaire- and task-based measures (the COVID-Dynamic study: 20). Across multiple waves, this study collected data from the same participants as national and world events unfolded (Fig. 1*A*; data are publicly available, see *SI Appendix*, section 5.1 for resources). The present investigation combines assessments of participants’ emotional state collected from April to December 2020, with memory-specific measures collected from the same participants in December 2020, as well as 1 y later (which we compared to memory for 2021), and again 2 y later (which we compared to memory in a new, naive sample of participants).

We identified the temporal structure of autobiographical memory by comparing the number of memory entries for each month of 2020 (retrieved in December 2020, and again 1 and 2 y later) to that for each month of 2021. We predicted and preregistered (22) greater and earlier spontaneous recall for March 2020, during the onset of COVID-19 emergency and lockdown announcements, than any other month, while we did not expect this pattern for 2021 memory. Given that a longitudinal sample, with repeated memory testing and associations with the COVID-Dynamic study, could potentially bias results, we recruited a naive sample of participants during our third data collection and recorded their memories of 2020 (all relevant findings were replicated, reported in *SI Appendix*, section 3.1).

We quantified the emotional and mnemonic content of autobiographical memory using several Natural Language Processing (NLP) tools. First, to provide a descriptive summary, we performed topic modeling on each memory collection (full procedure and results: *SI Appendix*, section 2.3.1; 21). Memory for 2020 included topics relevant to both personal and real-world events (Fig. 1*B*), which we observed across all memory sets (*SI Appendix*, Fig. S2).

We further analyzed memory content by using an NLP tool that classified the number of episodic-like or “internal” (versus “external”) details (as defined by the Autobiographical Interview: *SI Appendix*, section 2.3.2; 23). Here, we expected that higher recall for March 2020 would include episodic details, as unexpected events or changes in context (i.e., “event boundaries”) engage hippocampal memory systems, responsible for the formation of rich, event-specific, memory (24, 25). We also verified the number of described events through GPT-3 event segmentation (*SI Appendix*, section 2.3.4; (26), further corroborated by human episodic-event segmentation of a subset of autobiographical memories (*SI Appendix*, section 3.2.3).

Given that strong negative experiences are associated with accessible, vivid, and durable memories (27), we classified the sentiment of autobiographical memory (*SI Appendix*, section 2.3.3; (28) and expected negative affect, in particular, to modulate memory. To directly probe this relationship, we leveraged the battery of assessments collected from April to December 2020 and tested how self-reported affect during each month of 2020 predicted subsequent memory for that month. We hypothesized that stronger negative emotions experienced over a month would increase the likelihood of later retrieving events from that month. We also expected the degree of positive and negative experienced emotion to predict the positive and negative sentiment of subsequent memories for that month, respectively.

In addition to fluctuating month-to-month affect, we investigated how more stable individual differences influenced general properties of autobiographical memory. To this end, we conducted an exploratory and confirmatory factor analysis on our measures of affect and used the resulting factors, as well as markers of PTSD, to predict overall signatures of autobiographical memory. Here, we had two competing hypotheses: Given the strength of negative memory (27), higher overall negative affect could lead to more recall; alternatively, since chronic stress can impair memory (29, 30), it could lead to lower recall. Moreover, emotionally intense negative memory and rumination, such as in PTSD and depression, have been associated with “overgeneralized” memory, meaning there is less episodic specificity (i.e., more external versus internal detail) within autobiographical memory (31, 32). We therefore hypothesized that higher self-reported indices of depression and PTSD would predict less episodic memory.

While these autobiographical memories for 2020, a year marked by unprecedented events and transitions for many, provide a window into how collective events shift personal narratives, they do not necessarily constitute a direct measure of memory for collective events. We therefore conducted a study on a new sample of participants in 2022 and evaluated the memorability, affective response (including surprise, positive emotion, and negative emotion), as well as coarse temporal accuracy for the top news topics of every 2020 month. This study characterized the relationship between remembered affect and memorability of news events and provided an additional metric to assess the vividness of COVID-19 news. To note, we mean “collective memory” to simply refer to memory for collective events rather than the memory that comes to define a group’s shared identity (33). Nonetheless, we expect similar processes at play in the formation of shared memory across individuals.

Furthermore, as high prediction-error events (8) and collective transitions, such as COVID-19 lockdowns (18, 34), are likely to influence temporal memory (i.e., memory for the relative timing of events), we also included a measure of subjective time between 2020 news events. We had two competing hypotheses. If the pandemic acts like an event boundary in memory, thereby dilating perceived time (9, 35), events that crossed the boundary would be perceived as further away from each other than those that did not. On the other hand, lockdowns, predominant from March through May (Fig. 1*A*), have been theorized to produce a “dip” in autobiographical memory (18), as they restricted movement and accessibility to different contexts, precluding the formation of novel, distinct events in memory. For this reason, temporal memory may be compressed between events that crossed the lockdown period, leading to shorter estimates of subjective time. We evaluated these hypotheses by testing pairs of news events that either spanned the height of the lockdown period or did not.

## Results

### Greater Autobiographical Memory for March 2020 (Pandemic Onset) across 3 y.

**Fig. 2.**
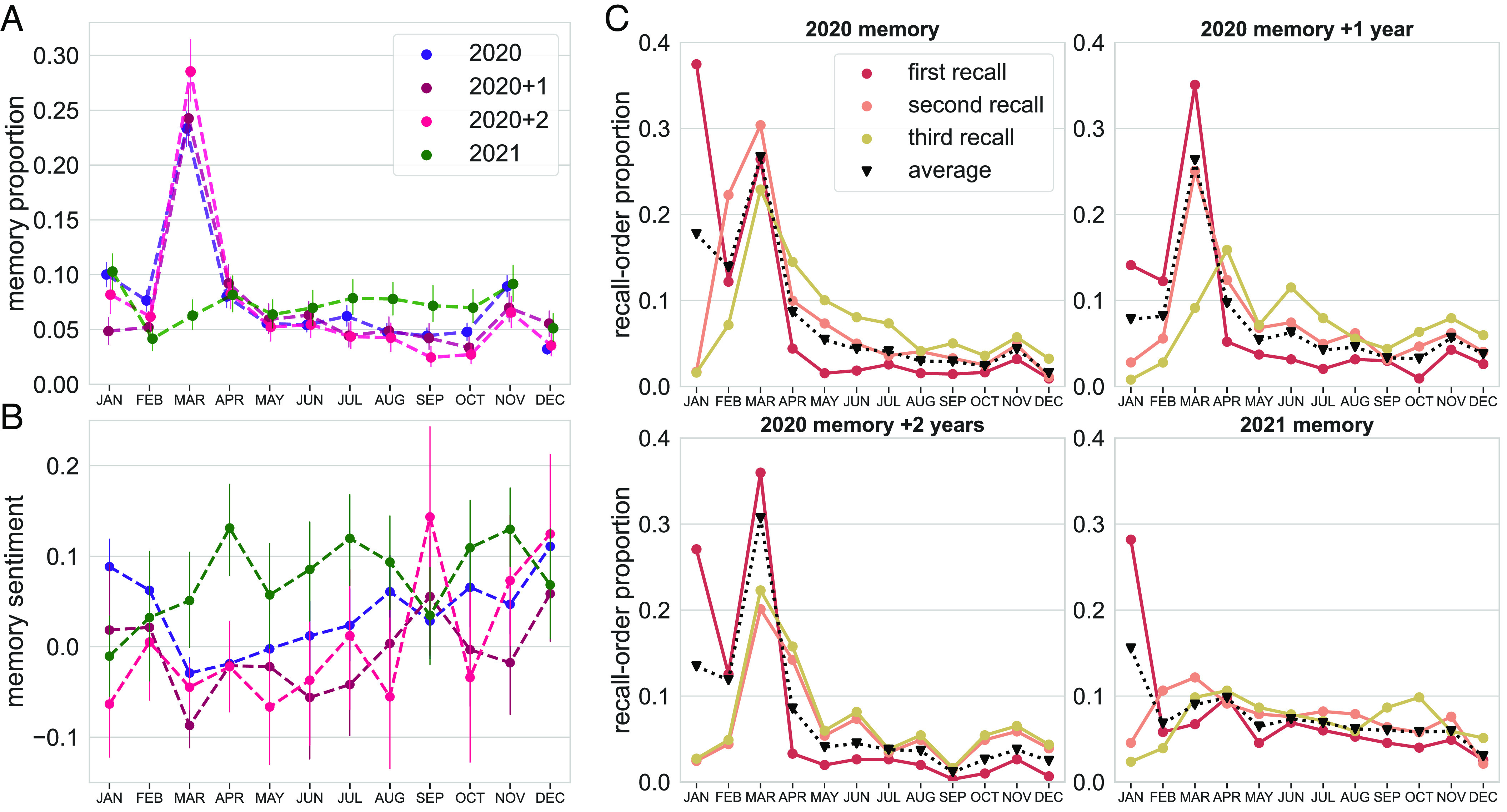
Distribution and characteristics of recollections by month. Participants first recalled events as they spontaneously remembered them and then dated each memory by month. (*A*). Mean across within-participant proportions (including all possible date options) of memory for each month. Memory for March 2020 was greater when recalled that year, as well as one (2020+1) and two (2020+2) years later; this pattern was not observed for memory of 2021. Colors indicate which year and when it was recalled (e.g., “2020+1” is memory for 2020 recalled 1 y later); error bars are SEM across subjects. Note that 2020 and 2021 memory collections occurred in early December of those years (i.e., recency effects are captured by November memory; for those collections, dotted lines between November and December are omitted). See *SI Appendix*, Fig. S4 for individual-level plots across all date options. (*B*). Average sentiment score for retrieved memories ranging from −1 (negative) to 1 (positive). Memory for March 2020 was more negative than other months of 2020; this was not the case for 2021 memory. Additionally, more negative sentiment predicted a greater amount of recall for that month, regardless of memory collection. (*C*). Proportion of participants’ dated recall for their first through third entries by memory collection; colors indicate recall number, black dotted line is the average across first three entries. In addition to the tendency to recall a year sequentially (i.e., higher proportion of January memory as the first entry), March was recalled earlier than other months of 2020; this pattern did not occur for 2021 memory.

We quantified the number and proportion of memory entries recalled for each year, broken down by month (visualized proportions show average across within-subject proportions; Fig. 2*A*). To ensure that this measure reflected the number of distinct episodic events, we implemented three additional approaches: 1) automatic scoring of the Autobiographical Interview for episodic (internal) content (using a trained NLP model (23); model details: *SI Appendix*, section 2.3.2), 2) GPT-3 event segmentation (using a predictive approach (26); model details: *SI Appendix*, section 2.3.4), and 3) human-rated episodic-event segmentation on a subset of autobiographical memories. We replicated all results using both 1) retrieved episodic details (*SI Appendix*, section 3.2.1), 2) GPT-3 event count (*SI Appendix*, section 3.2.2), and 3) further verified that memory count measures correlated with human episodic-event segmentation (*SI Appendix*, section 3.2.3).

We found a clear pattern: There was a pronounced peak for autobiographical memory from March 2020, both when recalled December of that year (memory-entry count as a function of month, mixed-effects Poisson regression: *β* = 1.07, *z* = 30.65, *P* < 0.001; M = 1.07 [1.00, 1.14]; Fig. 2*A*), 1 y later (*β* = 1.29, *z* = 23.93, *P* < 0.001; M = 1.29 [1.19, 1.40]), and 2 y later (*β* = 1.58, *z* = 27.96, *P* < 0.001; M = 1.58 [1.47, 1.69]; replicated in a new sample of participants: *SI Appendix*, section 3.1). To note, the March-2020 memory “bump” was stronger in the third memory collection than the first two (interaction between month and third-vs.-first collection in predicting memory count: *β* = 0.51, *z* = 7.65, *P* < 0.001; M = 0.51 [0.38, 0.64]; interaction between month and third-vs.-second collection: *β* = 0.29, *z* = 3.68, *P* < 0.001; M = 0.29 [0.13, 0.44]), suggesting that March-2020 memory was protected from memory decay.

On the other hand, recall for March 2021 was not greater than other months in 2021 (*β* = −0.08, *z* = -1.03, *P* = 0.30; M = −0.09 [−0.26, 0.07]); this effect was significantly different from that for 2020 memories even when both were collected at the end of 2021 (interaction between month and year recalled: *β* = −1.16 *z* = −12.92, *P* < 0.001; M = −1.16 [−1.34, −0.98]). The pattern we observed for monthly recall is consistent with the hypothesis that memories were preferentially stored during times that were the most surprising and life changing: the onset of the COVID pandemic.

### Primacy and Recency Effects.

There was also evidence for classic primacy and recency effects (36) when recalling autobiographical memory for the present year (i.e., 2020 memory at the end of 2020 and 2021 memory at the end of 2021). There was greater recall for the first month of the year, January, compared to February (*β* = 0.40, *z* = 6.94, *P* < 0.001; M = 0.40 [0.28, 0.51]; Fig. 2*A*), and for the most recent month, November (data were collected early December) compared to October (*β* = 0.52, *z* = 8.46, *P* < 0.001; M = 0.52 [0.40, 0.64]). We note that recency memory for November 2020 may be confounded with memory for the presidential elections (Fig. 1*B*); nevertheless, we observed a recency effect for November 2021 without such an external confound.

### Earlier Recall of March-2020 Memory.

Given that stronger, more accessible memory is recalled first (37, 38), we next characterized the order of spontaneous recall. We visualized the first through third memory entries of each subject and plotted the proportion of entries belonging to each month (Fig. 2*C*). Although there was an expected peak of first recall for January, likely due to this simply being the start of the year from which participants were asked to retrieve memories, there was again a pronounced peak for March 2020 as one of the first recalled memories. Memories from March 2020 were retrieved earlier when recalled in December 2020 (*β* = −2.00, *t* = −15.34, *P* < 0.001; M = −2.00 [−2.26, −1.74]), 1 y later (*β* = −1.70, *t* = −11.37, *P* < 0.001; M = −1.70 [−2.00, −1.41]), and 2 y later (*β* = −1.90, *t* = −9.97, *P* < 0.001; M = −1.90 [−2.28, −1.52]; also replicated in a new sample of participants: *SI Appendix*, section 3.1). During 2021 recall, March was also retrieved earlier, which, given March’s early position in a year, was anticipated (*β* = −0.97, *t* = −3.79, *P* < 0.001; M = −0.97 [−1.48, −0.46]). Nevertheless, this effect was not as strong as it was for 2020 memory (interaction between month and year recalled: *β* = 1.03, *t* = 3.39, *P* < 0.001; M = 1.02 [0.43, 1.60]), suggesting unique accessibility of March 2020 memory.

### March-2020 Memory Contained More Negative Content That Predicted Greater Recall.

We used a well-established NLP tool to analyze the sentiment of autobiographical memory (VADER, *SI Appendix*, section 2.3.3; 28). The sentiment analysis (ranging from −1, negative sentiment, to 1, positive sentiment) revealed that memories for March 2020 were more negative than other months, when recalled in December 2020 (*β* = −0.07, *t* = −5.62, *P* < 0.001; M = −0.07 [−0.10, −0.05]; Fig. 2*B*), 1 y later (*β* = −0.08, *t* = −4.39, *P* < 0.001; M = −0.08 [−0.12, −0.05]), and 2 y later (*β* = −0.04, *t* = −1.92, *P* = 0.056; M = −0.04 [−0.09, −0.001]; also replicated in a new sample of participants: *SI Appendix*, section 3.1). On the other hand, memory for March 2021 was not more negative than other months of 2021 (*β* = −0.03, *t* = −1.13, *P* = 0.26; M = −0.03 [−0.09, 0.02]).

Furthermore, we found, across all autobiographical memory (treating memory collection as a random effect), that more negative sentiment was associated with greater recall for the corresponding month (*β* = −0.08, *z* = −3.13, *P* = 0.002; M = −0.09 [−0.15, −0.04]). Given that strong emotions (regardless of valence) modulate memory (27), we further separately tested the strength of positive and negative sentiment on the amount of recall. We found each to be significant, and in the same direction of sentiment: less positive (*β* = −0.18, *z* = −2.28, *P* = 0.02; M = −0.18 [−0.34, −0.02]) and more negative sentiment (*β* = 0.38, *z* = 4.10, *P* < 0.001; M = 0.38 [0.19, 0.56]) both predicted more recall. This finding points to an overall relationship between negative memories and greater recall. We next directly probed this mechanism by investigating whether experienced affect during a month of 2020 predicted later autobiographical memory for that month.

### Negative Affect Increased Likelihood of Later Retrieving That Month.

**Fig. 3.**
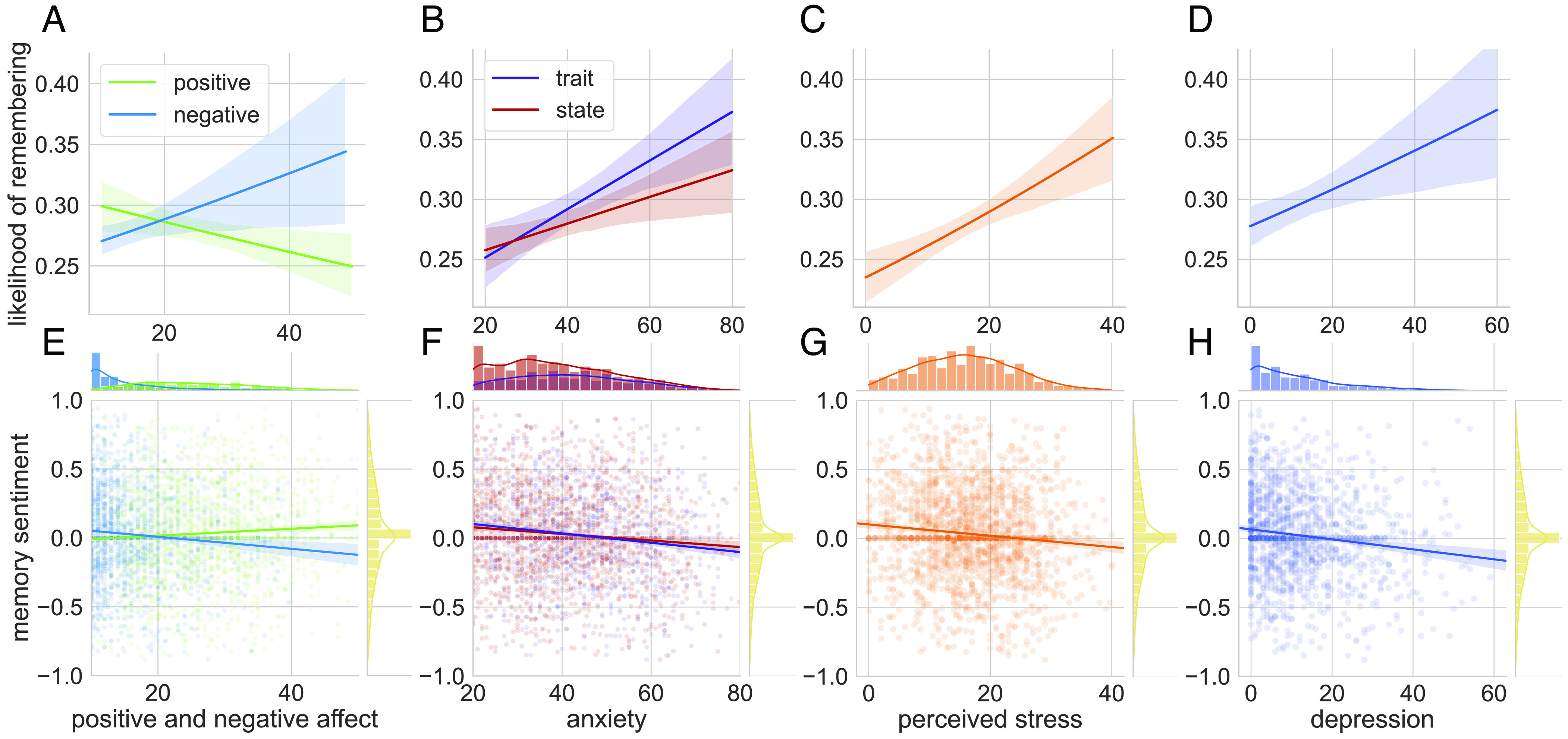
Likelihood of later retrieving a month and its sentiment given experienced emotion during that month. We administered a series of assessments from April to December of 2020 that evaluated immediate and longer-term affect and tested whether these measures predicted the likelihood of that month being later remembered in autobiographical memory (*A*–*D*). We found that negative affect, as assessed by (*A*) immediate negative affect (39), (*B*) state and trait-level anxiety (40), (*C*) experienced stress (41), and (*D*) depression (42), increased the likelihood of later recalling that month. Shaded regions reflect 95% CIs. (*E*–*H*). We also found that all measures predicted the sentiment of autobiographical memories (ranging from −1, negative, to 1, positive) in the expected direction: (*E*) immediate negative affect led to more negative sentiment for that month, while immediate positive affect led to more positive sentiment; (*F*) increased state and trait-level anxiety, (*G*) stress levels, and (*H*) depression also predicted more negative sentiment for that month. Shaded regions reflect 95% CIs. Marginal distributions represent the variable on the corresponding axis.

We tested each measure of interest (see “Monthly measures” in *Materials and Methods*) for whether average self-reported affect during a month of 2020 predicted whether that month was later reported in autobiographical memory. We found that both short- and longer-term negative affect led to a greater likelihood of remembering that month (Fig. 3 *A–D*; full results table: *SI Appendix*, Table S3). This included immediate negative (and not positive) affect (Fig. 3*A*), state and trait-level anxiety (Fig. 3*B*), stress (Fig. 3*C*), and depression (Fig. 3*D*). We did not find resilience nor the (face-icon) mood measure to affect the likelihood of recalling that month.

### Affect Influenced Sentiment of Retrieved Memory.

We also investigated whether experienced emotions influenced the expressed sentiment of that memory (sentiment analyzer: *SI Appendix*, section 2.3.3). All tested measures predicted the valence of later memory for that month in the predicted direction. Specifically, immediate negative affect (Fig. 3*E*), negative mood, state and trait-level anxiety (Fig. 3*F*), stress (Fig. 3*G*), and depression (Fig. 3*H*) led to more negatively valenced memory for that month (full results table: *SI Appendix*, Table S4). On the other hand, self-reported positive affect (Fig. 3*E*) and resilience led to more positively valenced memory for the retrieved month.

### Six Factors Predicted the Amount and Sentiment of Recall.

We furthermore tested overall individual differences in affect (versus month-to-month fluctuations) on general properties of autobiographical memory. We performed a factor analysis to reduce the dimensionality of the different, but related affective scales tested above (for procedural details, see *SI Appendix*, section 2.2). The exploratory factor analysis (run on all COVID-Dynamic participants during one wave of data collection) revealed six factors that accounted for 56% of the variance in the data (each ranged from 7% to 11%); rms error = 0.03, rms error of approximation = 0.055 [0.054, 0.056]. Supported by a confirmatory factor analysis (run on a different wave of data collection; *χ*^2^(3321) = 68,526.00, *P* < 0.001), individual items loaded onto six interpretable factors (for item loadings, see *SI Appendix*, Fig. S1): positive low-arousal (e.g., “ease,” “content,” and “relaxed”), negative low-arousal (e.g., “fatigue,” “loss of energy,” and “loss of interest”), positive high-arousal (e.g., “enthusiastic,” “excited,” and “active”), negative high-arousal (e.g., “scared,” “upset,” and “frightened”), positive self-image (e.g., “can deal with life,” “can adapt,” and “strong person”), and negative self-image (e.g., “failure,” “worthlessness,” and “inadequate”).

**Fig. 4.**
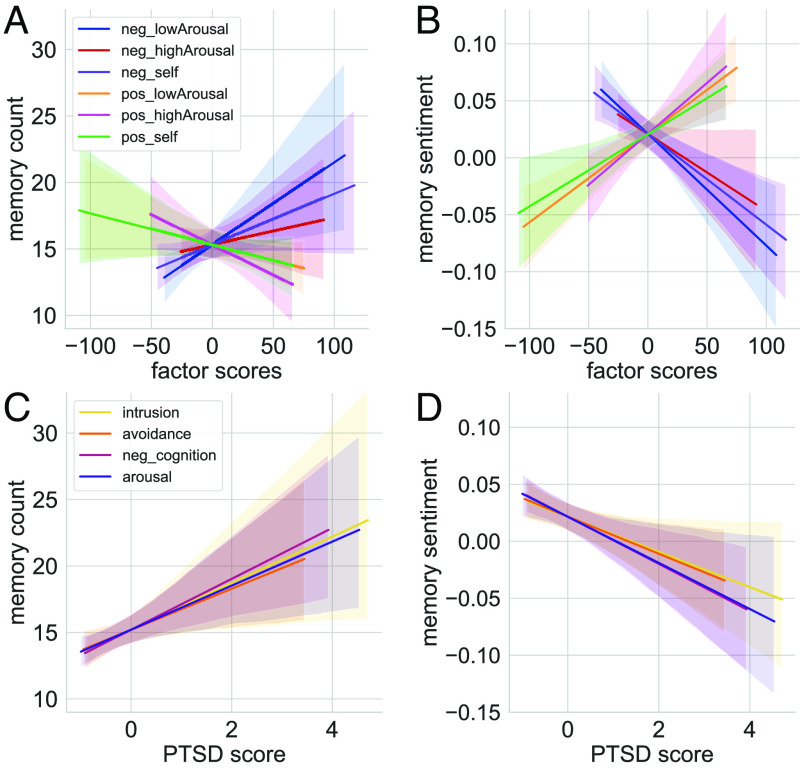
Individual differences in affect and PTSD predict autobiographical memory. (*A* and *B*). We conducted an exploratory and confirmatory factor analysis on all affect measures and found six meaningful factors that varied on valence (positive or negative), arousal (low or high), and relevance to self: negative low-arousal, negative high-arousal, negative self, positive low-arousal, positive high-arousal, and positive self (colors represent each factor). We found that negatively valenced factors predicted greater amount of recall and more negative sentiment in autobiographical memory, whereas positively valenced factors predicted the opposite. (*C*–*D*). We also found that PTSD symptoms (memory intrusion, avoidance of trauma-related thoughts, negative cognition, and hyperarousal; 43) led to more recall (although specifically for nonepisodic or external details, see the main manuscript) and more negative sentiment in autobiographical memory (colors represent each symptom, measures were each z-scored for visual comparison). Shaded regions reflect 95% CIs.

We multiplied participant ratings for the 82 items by each factor’s loading score and calculated average subject-level factor scores. We then tested whether these individual differences predicted the overall number and average sentiment of people’s memories. We indeed found that the three negative factors were associated with a greater amount of recall (Fig. 4*A*; full results table: *SI Appendix*, Table S5), as well as more negatively valenced memories (Fig. 4*B*; full results table: *SI Appendix*, Table S6). On the flip side, the three positive factors predicted less recall (Fig. 4*A* and *SI Appendix*, Table S5) and more positively valenced memories (Fig. 4*B* and *SI Appendix*, Table S6).

We also evaluated whether these six factors predicted the overall number of internal (episodic) and external (nonepisodic) details used to describe memory (*SI Appendix*, section 2.3.2). We found, using robust linear regression (to account for the influence of outliers, *SI Appendix*, section 2.1), that positive high-arousal predicted less internal detail (*β* = −6.44, *t* = −2.19, *P* = 0.03; M = −6.99 [−12.12, −1.74]), while negative low-arousal (representing depression items that are less related to self-cognition, *SI Appendix*, Fig. S1) predicted more external (nonepisodic) detail (*β* = 6.89, *t* = 2.38, *P* = 0.02; M = 6.17 [0.58, 11.89]).

### PTSD Predicted Greater and More Negative Memory, Especially for Nonepisodic Details.

Finally, given the powerful effects traumatic memory has in PTSD, we investigated whether self-reported PTSD symptomatology (DSM-5: 43) modulated the amount and content of memory. We calculated average participant scores for each of the four subscales (symptom clusters) of the PTSD checklist: 1) memory intrusion, 2) avoidance (of trauma-related thoughts), 3) negative cognition, and 4) hyperarousal. Every measure of experienced PTSD predicted greater amount of recall (Fig. 4*C*; full results table: *SI Appendix*, Table S7) and more negative sentiment (Fig. 4*D*; full results table: *SI Appendix*, Table S8).

Moreover, given that PTSD is marked by less episodic specificity in autobiographical memory (31), we tested whether symptoms predicted the amount of external or internal detail. We did not find any PTSD symptom to reliably influence the number of internal (“episodic”) detail; however, strikingly, every symptom predicted more external (“nonepisodic”) detail (intrusion: *β* = 10.07, *t* = 3.59, *P* < 0.001; M = 8.77 [2.98, 14.55]; avoidance: *β* = 10.07, *t* = 3.66, *P* < 0.001; M = 10.33 [4.81, 15.88]; negative cognition: *β* = 10.48, *t* = 3.76, *P* < 0.001; M = 9.80 [4.18, 15.53]; arousal: *β* = 8.57, *t* = 3.04, *P* = 0.003; M = 7.18 [1.73, 12.62]). This finding is consistent with previous work showing memory “overgeneralization” in PTSD, where retrieval for specific events or episodic detail is impaired (44).

### Collective Memory for Pandemic News Was Stronger, More Surprising, and Negative.

**Fig. 5.**
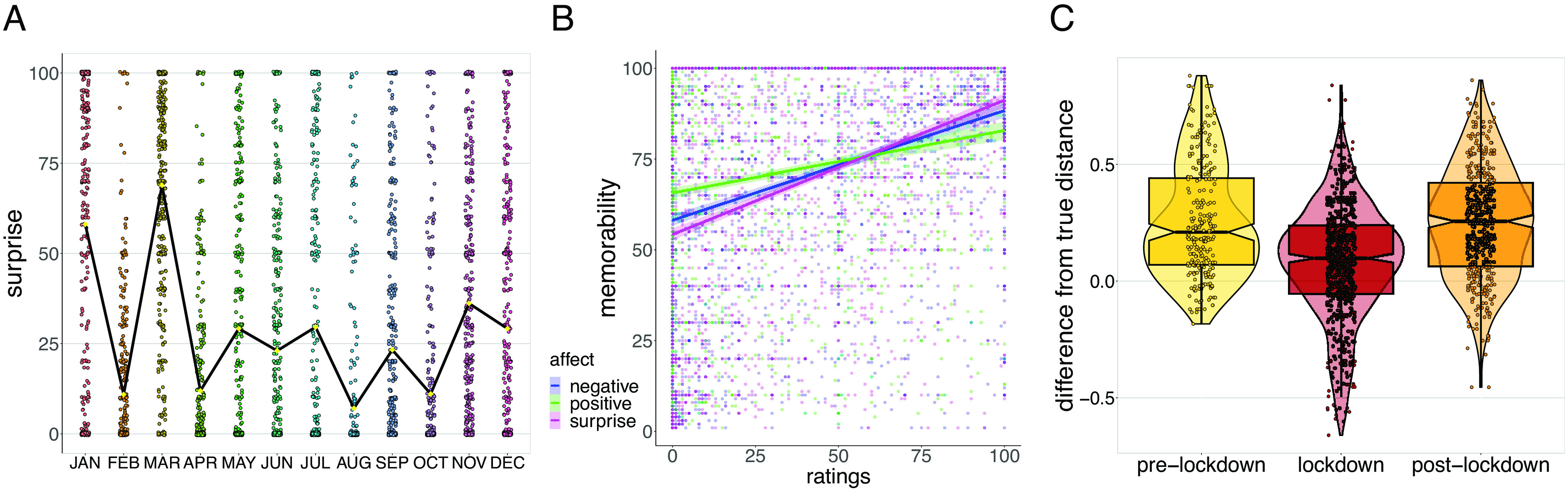
Collective and affective memory for top 2020 news events and estimated time between them. We asked a new cohort of participants in 2022 to rate the two top 2020 news topics for each month on memorability and remembered affect (ratings ranged from 0 to 100). (*A*) Surprise ratings for news events, averaged by month (emergency COVID-19 news included in March), yielding a similar pattern to the distribution of 2020 autobiographical memory. (*B*) The strength of remembered surprise, negative affect, and positive affect were each independently associated with greater memorability of retrieved news items. (*C*) Participants also estimated how close or far apart pairs of news events were in time (from 0, very close, to 100, very far); some pairs spanned the predominant lockdown period in the United States (Fig. 1*A*), and some did not. Controlling for actual distance, we found pairs that spanned the lockdown were judged as closer to each other than pairs that did not (there were no differences between pairs that occurred pre- and postlockdown).

To verify the memorability and emotional response (surprise, positive affect, and negative affect) to collective events, we recruited a separate sample of participants to date and rate the top two news events of each month of 2020 (determined by Google Trends, *SI Appendix*, section 1.2). We verified that emergency news of COVID-19 in March was more memorable and more accurately dated than other remembered events (i.e., when excluding not-remembered events; memorability: *β* = 0.81, *t* = 12.64, *P* < 0.001; M = 0.81 [0.68, 0.93]; error: *β* = −0.61, *t* = −8.51, *P* < 0.001; M = −0.61 [−0.74, −0.47]). Moreover, this news was associated with higher surprise (*β* = 1.06, *t* = 15.94, *P* < 0.001; M = 1.06 [0.93, 1.20]), confirming that it was a strong collective prediction-error event (Fig. 5*A*). Participants also rated their affective reaction to COVID-19 news as more negative and less positive (negative: *β* = 1.01, *t* = 14.47, *P* < 0.001; M = 1.01 [0.87, 1.14]; positive: *β* = −0.51, *t* = −7.05, *P* < 0.001; M = −0.51 [−0.65, −0.36]), in line with the increased negative sentiment associated with March 2020 autobiographical memory (news items and average ratings: *SI Appendix*, Table S1).

### Remembered Surprise, Positive Affect, and Negative Affect Were Separately Associated with Greater Event Memorability.

Moreover, across all remembered news items, we found that the degree of surprise, negative affect, and positive affect each independently predicted greater memorability (all variables tested in a single regression model, surprise: *β* = 0.23, *t* = 15.70, *P* < 0.001; M = 0.23 [0.20, 0.26]; negative affect: *β* = 0.52, *t* = 30.63, *P* < 0.001; M = 0.52 [0.48, 0.55]; positive affect: *β* = 0.45, *t* = 28.30, *P* < 0.001; M = 0.45 [0.42, 0.48]; Fig. 5*B*), consistent with our expectation that more experienced (and in this case remembered) affect is associated with more memorable news events.

### Lockdown Compressed Perceived Time between Events.

We lastly asked participants to estimate the subjective distance between pairs of 2020 news events (from 0 “very close” to 100 “very far”). To verify a degree of event knowledge, we only analyzed pairs of news events that were both remembered and correctly (relatively) ordered in time (*SI Appendix*, section 1.2). First, we confirmed that actual distance between events predicted subjective distance (*β* = 0.16, *t* = 17.82, *P* < 0.001; M = 0.16 [0.14, 0.17]) and that memorability ratings (for retrieved items) did not have further effects (*β* = −0.003, *t* = −0.082, *P* = 0.94; M = −0.00 [−0.09, 0.08]). We then computed a measure accounting for the actual distance between events (“difference from true distance”). Here, true (normalized) distance was calculated by dividing the number of days between two events by the number of days in a year (365). We then subtracted this from subjective (normalized) distance, calculated by dividing participant estimates by the scale’s range (100). For example, let us say two events were 90 d apart and a subject estimated that the events were temporally close by clicking “10” on the scale. We would then subtract (90/365 =) 0.24 from (10/100 =) 0.10, which equals −0.14, meaning that the two events were estimated as closer to each other than their actual distance within the year.

We found that while tested pairs of news events crossing the emergency COVID-19 announcements and lockdowns were, on average, actually more days apart (166 d) than pairs before (55 d) or after it (114 d), they were estimated as subjectively closer in time (lockdown versus prelockdown: *β* = −0.17, *t* = −9.83, *P* < 0.001; M = −0.17 [−0.21, −0.14]; lockdown versus postlockdown: *β* = −0.17, *t* = −11.71, *P* < 0.001; M = −0.17 [−0.20, −0.14]; Fig. 5*C*). There was no difference in perceived distance between events that occurred before versus after the lockdown period (*β* = −0.002, *t* = −0.08, *P* = 0.93; M = −0.00 [−0.04, 0.03]).

We also compared raw subjective-time estimates between a subset of pairs that were approximately the same time apart (2 to 3 mo), further controlling for actual days apart as a regressor in statistical models. While tested lockdown pairs were again, on average, actually more days apart (75 d) than pairs occurring before (55 d) or after it (51 d), we found that they were perceived as closer to each other in time (lockdown versus prelockdown: *β* = −10.93, *t* = −3.90, *P* < 0.001; M = −10.85 [−16.56, −4.97]; lockdown versus postlockdown: *β* = −15.11, *t* = −4.55, *P* < 0.001; M = −15.11 [−21.75, −8.64]); we did not find a significant difference between pairs that occurred before versus after lockdown (*β* = 4.18, *t* = 1.81, *P* = 0.07; M = 4.18 [−0.43, 8.79]).

## Discussion

The dynamic between an individual and their external world is profoundly captured in memory. The developing field of collective memory characterizes mechanisms that drive the mnemonic interaction between an individual and the events that come to define their collective history (45–48). This includes work replicating classic laboratory findings, such as primacy and recency effects (36), onto real-world historical memory (49). Here, we investigated whether the collective prediction error provoked by the COVID-19 pandemic, reaching emergency status in March of 2020, reproduced laboratory evidence of prediction-error memory enhancement (1, 27). Memory for March 2020 was indeed remembered more, evidenced by its increased and earlier retrieval across three timepoints (each 1 y apart) and replicated in a separate sample, demonstrating generalizable and stable long-term effects. Although the ecological nature of these data may not allow for separate testing of encoding, consolidation, and rehearsal mechanisms in improving memory, we expect each to have contributed to the marked, enduring, and, if anything, increasing March-2020 memory bump over time.

While this enhancement could be likened to historical “flashbulb” memories for unexpected events (13–15, 50), the pandemic ushered considerable changes to daily life, which are thought to have a uniquely strong influence on autobiographical memory (17). In fact, the extent of change, operationalized in recently proposed “Transition Theory”(18), is hypothesized to modulate the distribution of autobiographical memory, further predicting that the initial instability of the pandemic would lead to the bump in autobiography we observed in our data. More broadly, studies of autobiographical memory consistently observe “reminiscence bumps” for positive transition periods across a lifespan, which can vary in expectedness (51). In an exploratory individual-difference analysis (*SI Appendix*, section 5.2), we found some evidence of this such that self-reports of “positive change” predicted relatively more memory for March. This effect, however, was additive rather than explanatory, that is, it increased March memory in individuals already demonstrating a March-memory bump. March-2020 memory itself was in fact more negative in sentiment, corroborating work demonstrating increased autobiographical memory for negative and unexpected events (52, 53). These findings therefore point to the influence of unexpected, high-impact transitions, regardless of whether they were personally negative or positive, in creating collective bumps in autobiographical memory.

These results further suggest that the collective structure of memory or “schema” can shape our personal, autobiographical schema, potentially by providing a scaffolding onto which we place our personal stories. While collective memory work is nascent in cognitive neuroscience (33), a recent study showed that collective memory schemas engage the same medial prefrontal circuitry as individual schemas (46), encouraging future experiments that directly probe distinctions or interactions between the two. Nevertheless, reliable links between collective and autobiographical memory may require external events as exceptional and impactful as the COVID-19 pandemic.

We additionally tested the structure of 2020 memory through subjective estimates of time. Large prediction errors and shifts in context (i.e., “event boundaries”) influence the organization of memory by disrupting associations between events that crossed them. Such boundaries (e.g., transition periods in our autobiography) can draw a cognitive map of our experience and, similar to spatial anchors (54), provide landmarks that help us navigate our memories. Remembered time can reflect the cognitive distance between events in such a map, dilating subjective time between segmented events, a process thought to rely on the hippocampus (9, 35, 55). The idiosyncrasies of the COVID-19 pandemic brought forward two competing hypotheses: 1) the high prediction-error onset of the pandemic would disrupt mnemonic representations between events that occurred before and after it, leading to larger or dilated estimates of time; alternatively, 2) the lack of context change during the lockdown period would hinder the formation of new and distinct memories, leading to smaller or compressed estimates of time for events spanning it.

We found support for this second hypothesis: Remembered events that crossed the lockdown period, across small and large actual distances, were perceived as closer to each other. While there are many potential mechanisms supporting mnemonic judgment of time (56, 57), we propose that restricted movement and fewer context changes led to fewer samples of unique events in memory, thereby compressing estimates. This finding is also consistent with Transition Theory, where the decrease in everyday change during the lockdown was predicted to generate a “dip” in autobiographical memory (18). While we did not observe this dip in the distribution of autobiographical memory during the height of the lockdown, spontaneous autobiographical recall may be too coarse a measure to differentiate pandemic-onset enhancement from lockdown impairment in memory. Estimates of time in long-term memory are to be further distinguished from, but should be compared to, online estimates collected during lockdown. A new database focused on subjective temporalities during the pandemic in fact highlights associations between objective and perceived isolation on the felt passage of time (34). Future studies can thus leverage data collected during lockdown to characterize relationships between online and long-term memory for elapsed time.

Across our analyses, we tested four types of affect measures on memory for 2020: 1) the sentiment of autobiographical memory, 2) month-to-month measures of immediate and longer-term emotion from April to December 2020, 3) individual differences in affective state as determined by a factor analysis across longitudinal questionnaires (yielding six underlying factors varying in valence, arousal, and relation to self), and 4) remembered emotional response to 2020 news events. Overall, we consistently found stronger negative affect to predict enhanced memory, in line with a large literature demonstrating the benefits of strong negative emotion on the encoding, consolidation, rehearsal, and retrieval of those events (27). On the other hand, we found higher positive affect, both in the sentiment of autobiographical memory and in our individual factors, to predict fewer autobiographical memories on average. These results could suggest the greater strength of negative affect on memory or, alternatively, could reflect a sampling bias whereby 2020 was marked by the heightened prevalence of negative emotion (58). Nevertheless, we still found that remembered positive emotion predicted a relatively stronger March-memory bump in autobiographical memory (*SI Appendix*, section 5.2) and greater memorability across all 2020 news events, consistent with the reported beneficial effects of strong positive emotion on memory (27).

Our monthly and individual-difference measures of affect also predicted memory content. As expected, both monthly measures and individual differences in positive and negative affect led to positively and negatively valenced memory, respectively. Our individual-difference results further demonstrated that positive high-arousal was associated with fewer internal (episodic) details overall, while negative low-arousal was associated with more external (nonepisodic) details overall. These findings suggest that individuals who score high on positive high-arousal are less likely to encode or retrieve episodic detail in memory. On the other hand, individuals who score high on negative low-arousal, which is largely made up of depression-related items, may be more likely to ruminate, providing memory that is nonepisodic in nature, in line with prior studies (32).

Moreover, while unexpected or stressful events can enhance memory through multiple mechanisms (e.g., attention at encoding, consolidation, rehearsal, and retrieval; 59–61), chronic levels of arousal or negative affect can disrupt memory. Both PTSD and depression have been linked to memory biases for negative events (29, 62, 63), a mechanism that has been proposed to reinforce clinical symptoms. More devastating, however, is evidence that experienced trauma can predict hippocampal and amygdalar atrophy in both PTSD and major depressive disorder (MDD; 64, 65). Paralleling the pattern for negative low-arousal described above, we found that all PTSD symptoms (memory intrusion, avoidance of trauma-related thoughts, negative cognition, and hyperarousal) predicted more negative sentiment and a greater amount of recall for specifically external (nonepisodic) details, replicating results from a previous study on combat veterans with and without PTSD (31). This increased generation of nonepisodic details has been associated with “overgeneralized” memory, where retrieval for specific details is impaired, a hallmark of autobiographical memory in both PTSD and MDD (44). Given striking convergence between mechanisms that support episodic memory and future simulation (66), this deficit in episodic specificity predicts deficits in future thinking as well (31). Our findings corroborate previous work and encourage treatments that amplify and specify details for better, alternative futures (such as in episodic specificity inductions: 67).

One challenge we faced was to dissociate the influence of fluctuating month-to-month measures from stable individual differences. While results testing monthly effects were often consistent with individual-difference effects, our analyses were distinct. For example, our monthly variables predicted the likelihood of later retrieving that month, whereas factor scores indexing traits were tested on the overall amount of recall. Nevertheless, this does not preclude the role of stable individual differences in influencing monthly affect measures. In our dataset, not all questionnaires were administered at every wave nor at regular intervals, which prevented us from tracking affective change across time for some of our measures. Our findings nonetheless do demonstrate that both fluctuating monthly affect and stable patterns each influenced later autobiographical memory. Future studies could more precisely characterize how month-to-month changes within an individual interact with their general affect to predict autobiographical memory.

To conclude, collective events of 2020 reshaped memory by prioritizing the surprising and stressful events of the pandemic in our autobiographies and by calibrating our memory for time. Fluctuating monthly assessments of emotion as well as individual differences in affect further tuned the distribution and content of these memories. Strikingly and across multiple levels, negative affect increased recall, while its more chronic markers (e.g., PTSD), selectively increased nonepisodic recall. Taken together, these findings demonstrate an interplay between collective events and individual affect in the sculpting and telling of our life stories.

## Materials and Methods

### Open-Science Practices.

We preregistered the hypotheses and analysis for all studies (https://osf.io/bmequ). Data and code to fully reproduce all results and figures are available (https://github.com/ninarouhani/2023_RouhaniAdolphs). See *SI Appendix*, section 5.1 for publicly available data and resources from the COVID-Dynamic project at large.

### Participants.

The Covid-Dynamic study sampled a cohort of over 1,000 adult Americans longitudinally starting April 2020 (see Fig. 1*A* for first 15 waves) on the online platform Prolific. The full study protocol was approved by Caltech’s Institutional Review Board, and all participants provided informed consent prior to beginning each wave. We recruited participants from this sample for three autobiographical memory collections, the first time in December 2020 (N = 976: 529 female, age = 18 to 76), the second time in December 2021 (N = 550: 311 female, age = 18 to 77), and a third time in January 2023 (N = 303: 170 female, age = 19 to 79). During this third collection, we also recruited a new cohort of participants (N = 252: 141 female, age = 18 to 80), naive to the COVID-Dynamic study, to replicate our pattern of findings (*SI Appendix*, section 3.1). We excluded participants using the following criteria (specified in preregistration: (22)): Participants who 1) only entered one entry, 2) spent less time than the lowest 1% of time spent across participants in the first collection (2 min), or 3) provided less than the lowest 1% in word count across participants in the first collection (seven words); this led to the exclusion of 38 participants (final N for each memory collection: 1) N = 939, 2) N = 523, and 3) N = 303, N-replication = 252).

The collective memory study recruited a new sample of 200 participants (114 female; age = 19 to 68) from Prolific (COVID-Dynamic participants were excluded from recruitment) in October 2022. All participants correctly completed at least 2 of 3 attention checks.

### Autobiographical Memory Task.

Participants were asked to recall their memories (“events, experiences, and feelings”) for 2020 or 2021 as they came naturally to mind and without memory aids (instructions were identical across memory collections: *SI Appendix*, section 1.1). After typing in one memory, they were prompted to type another until they indicated that they had no other recollections to enter. Participants were then asked to date each entry by month (12 options: January through December). If they could not remember the exact month, they could choose approximate seasons (four options, e.g., “approximately fall”) or they could select “I do not remember.” Memories for 2020 were collected three times across 3 y from the COVID-Dynamic cohort (and from a replication sample during the third collection). Memories for 2021 were collected from the COVID-Dynamic cohort in December 2021.

### Collective Memory Task.

The task was composed of three sections that tested people’s memory for US news events. In the first section, participants saw a list of 24 news items composed of the two highest rising and distinct news topics of each month of 2020, assessed by Google Trends (procedure and stimuli: *SI Appendix*, section 1.2). This included events such as the death of Kobe Bryant in January, Coronavirus announcements and lockdowns in March, and election results in November. For each of these events (presented in randomized order), participants provided ratings for the four following questions, on a scale from 0 to 100: 1) How strongly do you remember this event? (a strong memory was defined as memory for when and where they were), 2) How surprising was this event to you? 3) How positive were your emotions? 4) How negative were your emotions? In the second section, participants were asked to retrieve the month (12 options: January to December) of remembered news items. In the last section, participants provided subjective distance ratings between pairs of news items (from 0, very close, to 100, very far). The pairs were selected to capture three time periods: prelockdown (three pairs), during lockdown (three pairs), and postlockdown (three pairs); we further included two longer-distance pairs that spanned pre- to postlockdown (see *S1 Appendix*, 1.2 for more information).

### Autobiographical-Memory Cleaning and Processing.

We first performed a spell check on the data and manually approved each change. We then tokenized data for NLP tools, meaning that entries were split into sentences or words. The output was reviewed for potential errors. On the tokenized data, we conducted three NLP analyses: 1) topic modeling using Gibbs Sampling Dirichlet Multinomial Mixture (*SI Appendix*, section 2.3.1; 21); 2) automatic scoring of the Autobiographical Interview (*SI Appendix*, section 2.3.2; 23); and 3) sentiment analysis (*SI Appendix*, section 2.3.3; VADER: 28). See *SI Appendix*, section 2.3 for procedural details, additional output and results.

### Monthly Measures.

The COVID-Dynamic study included a number of psychological measures including mood, personality, and personal experience collected from April to December 2020. We focused on measures capturing both short-term and longer-term affective states: 1) the Positive and Negative Affect Scales (39), 2) the State-Trait Anxiety Inventory (40), 3) Beck Depression Inventory (42), 4) the Perceived Stress Scale (41), 5) the Connor-Davidson Resilience Scale (68), and 6) a measure where participants indicated which schematic facial expression described how they were feeling “right now,” from negative to positive mood [“(face-icon) mood measure”]. We tested whether these six measures predicted the amount and content of later autobiographical memory. Note that not every measure was collected nor did every participant complete each wave. We calculated the average score for each available measure per month of 2020 (April through December 2020).

### Statistical Methods.

We use mixed-effects modeling (69) for null-hypothesis significance testing as well as Bayesian multilevel modeling (70) to generate estimates and confidence intervals for the effects at hand. For each statistical model, we first report results from significance testing and then from Bayesian modeling (for the identical model): We denote M as the mean of the posterior distribution and enter the 95% credibility interval (CI) in brackets. We considered an effect reliable only if the Bayesian CI did not span 0. See *SI Appendix*, section 2.1 for model details.

## Supplementary Material

Appendix 01 (PDF)

Dataset S01 (DOCX)

## Data Availability

All modeling code and output is publicly available, organized by the order of reported results (https://github.com/ninarouhani/2023_RouhaniAdolphs). Anonymized quantitative data/code have been deposited in github (https://github.com/ninarouhani/2023_RouhaniAdolphs) (71). All other data are included in the article and/or supporting information.
