# Supplementary material for: Collective events and individual affect shape autobiographical memory
Source: Proc Natl Acad Sci U S A. 2023 Jul 11;120(29):e2221919120. doi: 10.1073/pnas.2221919120 (PMC10629560; doi:10.1073/pnas.2221919120)
Supplement: Supplementary file 1 — Appendix 01 (PDF) [file pnas.2221919120.sapp.pdf]

PNAS

Supporting Information: Collective events and individual affect shape autobiographical memory

Nina Rouhani, Damian Stanley, COVID-Dynamic Team, Ralph Adolphs

Nina Rouhani: nrouhani@caltech.edu

This PDF file includes:

1. Materials

1.1 Autobiographical memory instructions 3

1.2 Collective memory stimuli 3

Table S1. Collective memory headlines and ratings 5

2. Methods

2.1 Statistical models 6

2.2 Factor analysis 6

Fig. S1. Six factors and their loadings 8

2.3 Natural language processing tools 9

2.3.1 Topic modeling 9

Fig. S2. Topic modeling for autobiographical memory 10

Table S2. Topics and keywords across memory sets 11

2.3.2 Autobiographical Interview analysis 12

2.3.3 Sentiment analysis 12

2.3.4 Event segmentation model 12

3. Replication analysis

3.1 Replication sample 13

Fig. S3. Comparison between longitudinal and replication sample 13

Fig. S4. Distribution of recollections across all possible date options 14

3.2 Supporting measures 14

3.2.1 Episodic content 14

3.2.2 GPT-3 event count measure 15

3.2.3 Human rater verification of episodic-event count 16

4. Results tables

Table S3. Negative affect increased likelihood of later retrieval, statistical results 17

Table S4. Affect influenced sentiment of retrieved memory, statistical results 17

Table S5. Six factors predicted the amount of recall, statistical results 18

Table S6. Six factors predicted the sentiment of recall, statistical results 18
Table S7. PTSD symptoms predicted the amount of recall, statistical results 19
Table S8. PTSD symptoms predicted the sentiment of recall, statistical results 19

5. Covid-Dynamic project

5.1 Description 20
5.2 Exploratory analysis 20
Fig S5. Self-reported positive impact predicted strength of March-2020 memory 21
5.3 Acknowledgments 21
Table S9. Author contribution table 22

6. **References** 23

1. Materials

1.1 Autobiographical memory instructions. For all three autobiographical memory collections, participants received the following instructions (when retrieving the year 2021, '2020' was replaced by '2021'):

> **Please tell us about your experiences during 2020.**
> Describe 2020 in terms of the events, experiences, and feelings that were personally important to you. Please do not use any memory aids like calendars or pictures and focus instead on the memories that come to you naturally.
>
> Enter your first memory into the text field below. When you are done writing one memory and have another, select "enter another memory" and then click the >> button to move on to the next page. If you do not recall any more memories, select "I am done" and move on to the next page. **Enter each memory on a separate page. Please provide complete sentences and be as detailed as you can.** If nothing comes to mind immediately, it is okay to take a minute and think.

1.2 Collective memory stimuli. For a relatively unbiased metric of popular news topics in the U.S., we implemented a predetermined procedure using Google Trends (queried September 7, 2022, 1). Google Trends is a widely used research tool that reliably captures changes in public interest which have, in turn, predicted human behavior across fields (2). For example, their search indices were used to forecast infection peaks during the COVID-19 pandemic among many other studies highlighting their predictive utility (3, 4). Google Trends summarizes search queries across a representative sample of people (across billions of daily users), normalized by the total number of queries for the specified geography and time range (score ranges from 0-100, representing a topic's proportion relative to all searched topics). We restricted geography to the United States and selected date ranges spanning each month of 2020. For each month, we collected the top 10 rising news topics (out of the 25 provided), which reflect news topics with the largest growth in search volume across that month. We then selected the two highest rising and distinct topics for each 2020 month (Table S1, below).

For the subjective distance measure, we further selected pairs of news items that varied in whether they spanned the pandemic lockdown or not. Specifically, we included the following events: *pre-lockdown*: the US drone strike, the death of Kobe Bryant; *early-lockdown*: COVID-19 pandemic and lockdowns, first stimulus check announcement; *late-lockdown*: the murder of George Floyd, BLM protests; *post-lockdown*: the death of Ruth Bader Ginsburg and the 2020 presidential elections. We selected 3 pairs spanning the pre- to early-lockdown period, 3 pairs spanning the early to late-lockdown period, and 3 pairs spanning the post-lockdown periods; we further added 2 longer-distance pairs that spanned the pre- to post-lockdown period (11 pairs in total). To note, we selected 3 pairs for each time period (rather than more) as we were constrained by the fact that March occurs relatively early in the year.

While we could not experimentally control for the actual distance between events across pairs, we did account for differences in our (normalized) distance measure. We further tested whether our overall findings replicated in a subsample of pairs that represented more similar time intervals (i.e., compared pairs spanning two to three months while also controlling for actual distance as a regressor in statistical models). To account for event memory, we only analyzed pairs of remembered events (as indicated by self-report) and those that demonstrated correct relative temporal memory (using the month-accuracy measure). For example, if a participant indicated that Kobe Bryant's death had occurred in January or February and that the COVID-19 announcements occurred March or later, this would indicate correct relative temporal memory. To note, we chose not to filter the pairs by correct retrieval of the exact month since there would be an accuracy bias for events that occurred in the middle of the month versus those that occurred at the very beginning or end of the month.

2020 Month	News Event	Date	Memorability	Surprise	Positive Emotion	Negative Emotion
**January**	*Iranian general Qasem Soleimani is killed in a US drone strike leading to fears of an impending war.*	01/03/20	33.510	19.915	14.950	28.430
	Kobe Bryant and his daughter die in a helicopter crash.	01/26/20	81.185	67.165	0.930	87.715
**February**	*New Hampshire Democratic primary: Bernie Sanders, Pete Buttigieg and Amy Klobuchar win delegates.*	02/11/20	21.880	5.775	21.665	10.755
	Nevada Democratic caucus: Bernie Sanders, Joe Biden and Pete Buttigieg win delegates.	02/22/20	21.880	6.620	21.485	11.080
**March**	*Coronavirus is declared a global pandemic and the US in a state of emergency leading to stay-at-home orders nation-wide.*	03/11/20	94.255	76.540	9.820	79.745
	The first stimulus check is issued by the Coronavirus Aid, Relief and Economic Security (CARES) Act.	03/27/20	75.355	7.220	76.240	58.005
**April**	*British prime minister, Boris Johnson, becomes sick with coronavirus.*	04/06/20	42.995	17.170	9.805	17.435
	The National Football League has its first virtual draft (rather than in-person) because of COVID.	04/23/20	12.395	4.475	8.540	6.415
**May**	*A Black man in Minneapolis, George Floyd, is killed by a police officer; a video recording of his death.*	05/25/20	89.515	83.840	2.440	54.145
	Celebrity Mary-Kate Olsen files for divorce from Olivier Sarkozy.becomes widely circulated.	05/27/20	4.530	1.775	1.515	4.180
**June**	*The Black Lives Matter movement significantly ramps up, igniting protests across the US and the world.*	06/01/20	84.245	36.620	42.390	40.130
	Teens on TikTok advocate to "free" Donald Trump's son, Barron, from the White House.	06/17/20	9.450	3.685	4.835	5.940
**July**	*Singer August Alsina reveals he had an affair with Jada Pinkett Smith.*	07/02/20	22.145	12.775	3.685	20.250
	Actress Naya Rivera dies in a boating accident.	07/08/20	35.815	32.150	0.490	38.930
**August**	*Broadcaster Thom Brennaman is suspended after homophobic slur.*	08/19/20	4.290	5.215	1.675	4.925
	Jerry Falwell Jr. resigns as head of Liberty University amid scandal.	08/24/20	12.685	8.985	6.820	9.255
**September**	*In an interview with Donald Trump, Bob Woodward reveals that Trump deliberately played down the threat of the COVID pandemic.*	09/15/20	30.980	40.210	2.720	11.705
	Supreme court justice Ruth Bader Ginsburg dies.	09/18/20	67.950	49.820	4.075	34.855
**October**	*New Yorker suspends Jeffrey Toobin for misconduct on Zoom call.*	10/19/20	14.475	9.165	3.410	13.420
	Joe Biden and Donald Trump engage in presidential debates.	10/22/20	67.800	33.025	28.800	8.710
**November**	*Joe Biden wins 2020 presidential election.*	11/07/20	90.570	22.605	62.545	44.945
	Donald Trump demands election recount and claims voter fraud.	11/07/20	84.015	66.280	9.140	27.320
**December**	*Alec Baldwin's wife, Hilaria, is exposed as having faked a Spanish identity.*	12/28/20	12.265	5.255	2.165	9.415
	The US federal public sends out second stimulus check.	12/29/20	75.845	8.300	74.925	48.915

Table S1. Collective memory headlines and ratings. Top two rising news topics for each month of 2020, their date, as well as average memorability, surprise, positive and negative affect ratings from a separate cohort of participants recruited in October 2022.

2. Methods

2.1 Statistical models. We ran mixed-effects models (R package: lme4, 5) for null-hypothesis significance testing as well as Bayesian multilevel models (R package: brms, 6) to generate estimates and confidence intervals for the effect at hand. In all models, we treated participant as a random effect for the intercept as well as the slope of each fixed effect. If the model did not converge, however, we incrementally simplified the subject-level random-effects (e.g., by taking out interactions, then the slope of each effect), until convergence was achieved. At the minimum, subject-level intercept was modeled as a random effect. To test the effect of individual differences, we used standard linear regression (without random effects). The type of regression used was contingent on the dependent variable: for memory count, we used Poisson regression; for the likelihood of retrieval, we used binomial logistic regression; for testing proportions, we used quasi-binomial regression, and for all other models, we used linear regression. We scaled all predictor variables to ease model convergence.

We moreover verified whether any model was inordinately influenced by outliers and checked model output with and without outliers. If outliers were determined to influence predictor significance, we ran robust regression models for null-hypothesis testing instead (R package: mass, 7) and specified the more robust Student's t-distribution in Bayesian regression models. We used this approach to characterize the effects of individual differences (6-factor model and PTSD) on the total number of external and internal details.

2.2 Factor analysis. We used the full sample of participants in the COVID-Dynamic study (8) to perform an exploratory and confirmatory factor analysis on all affect measures: (1) the Positive and Negative Affect Scales (PANAS, 9), (2) the State Trait Anxiety Inventory (STAI, 10), (3) Beck Depression Inventory (BDI, 11), (4) the Perceived Stress Scale (PSS, 12), (5) the Connor-Davidson Resilience Scale (RISC, 13), and (6) a measure where participants indicated which schematic facial expression described how they were feeling 'right now', from negative to positive mood ('(face-icon) mood measure'). See Fig. S1 below for questionnaires and individual items.

The exploratory factor analysis was run on data collected August 2020 ('wave 10'; N = 1029 participants and 102 questionnaire items in total; over 10 subjects per item ratio, as recommended, 14), and the factors were confirmed on data collected September 2020 ('wave 12'; N = 932); these two waves of data collection were the first two to include all measures of interest. We first checked and confirmed the adequacy of our data for factor analysis using Bartlett's test of sphericity (15), $\chi 2(5151) = 92454.65$, $p < 0.001$, and the Kaiser-Meyer-Olkin Measure of Sampling Adequacy, MSA = 0.98 ('great'; 16). In order to determine the number of factors, we visualized scree plots and ran parallel analysis (R package: 'nFactors', 16), and subsequently conducted exploratory factor analysis using oblique rotation (R package: 'psych',

17). We achieved ‘simple structure’ in the number of factors and items by using the following criteria (14): (1) items, at minimum, must load 0.30 or higher on any factor, (2) items should only load (0.30 or higher) on a single factor (in the same direction), and (3) factors should have more than three items. The factor analysis was repeated until all criteria were met, we repeated this procedure four times and eliminated 20 of the 102 items (resulting in 82 items; Fig. S1). We next performed confirmatory factor analysis on the resulting structure (R package: ‘lavaan’, 18).

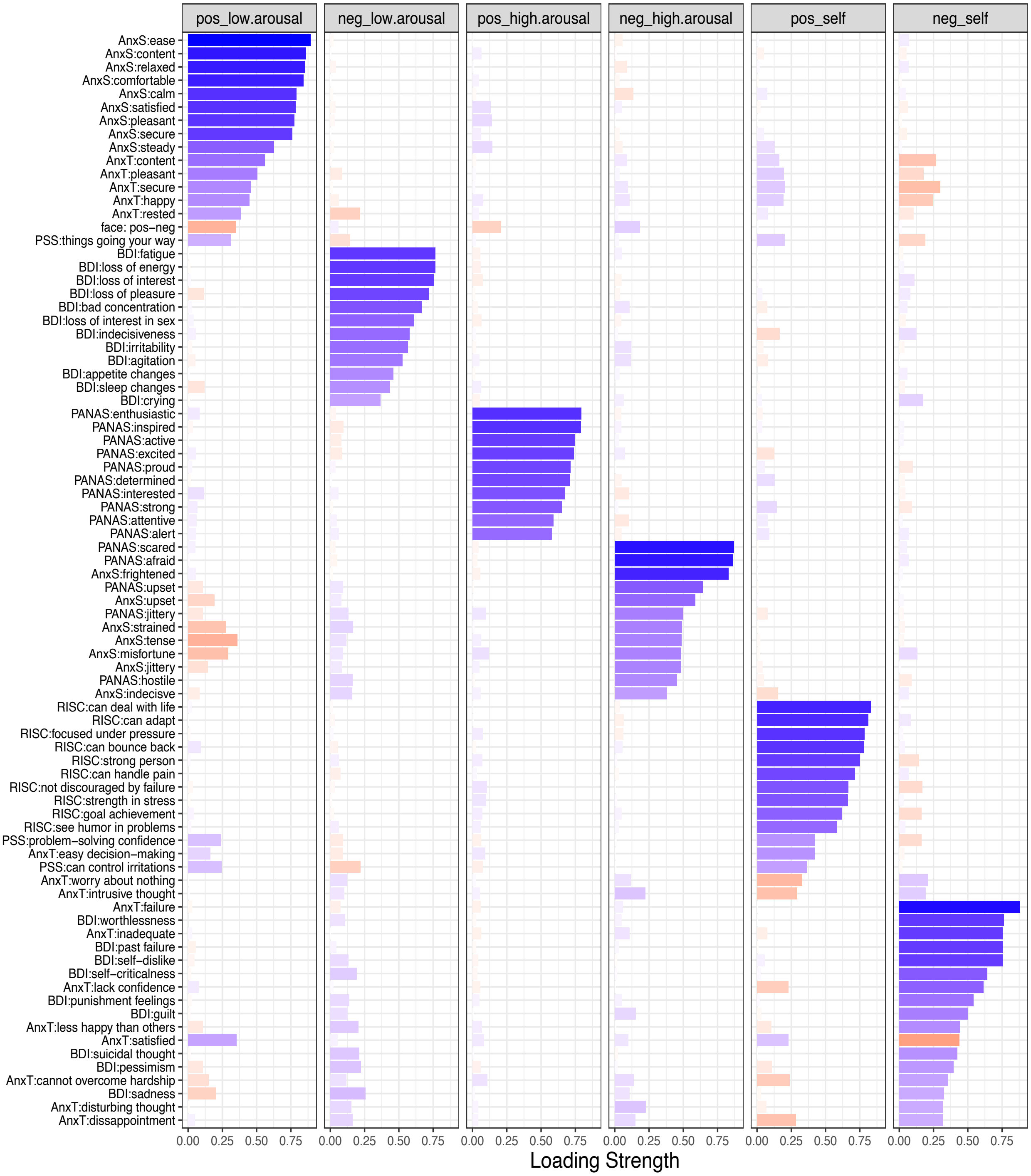

Fig. S1. Six factors and their loadings. We conducted an exploratory and confirmatory factor analysis on all items belonging to measures that assessed affect. Item loading strength varies from negative (orange) to positive (blue). Item notation on the y-axis includes scale (State Trait Anxiety Inventory (10): 'AnxS' – state, 'AnxT' – trait; Positive and Negative Affect Schedule (9): 'PANAS'; Perceived Stress Scale (12): 'PSS'; Beck Depression Inventory (11): 'BDI'; Connor-Davidson Resilience Scale (13): 'RISC'; (face-icon) mood measure: 'face') and brief description. The factor analysis revealed six meaningful factors that varied on valence (positive or negative), arousal (low or high arousal), and relevance to self: negative low-arousal, negative high-arousal, negative self, positive low-arousal, positive high-arousal, positive self.

2.3. Natural language processing tools

2.3.1 Topic modeling. In order to preprocess the data for topic modeling, we followed additional standard procedures on all memory entries in the following order: (1) punctuation removal, (2) word tokenization, (3) stop-word removal, (4) lemmatization, and (5) n-gram conversion (bigrams and trigrams) using Python's Natural Language Toolkit (NLTK, 19). After preprocessing, we reviewed the generated tokens for proper nouns that referred to the same event or person but were expressed by different variants and assigned them to the same token (e.g., variants of 'COVID-19' were all assigned to 'covid'). We further removed common words arbitrarily used in prose (or in the act of remembering) that were also associated with several (at least three) generated topics within any memory set: 'remember', 'memory', 'day', 'week', 'year', 'time', 'go', 'come', 'get', 'take', 'make', 'see', 'one', 'many', 'would', 'could', 'like', 'thing', 'us', 'start', 'first', 'also'.

As our topic model, we implemented Gibbs Sampling Dirichlet Multinomial Mixture (GSDMM, 20), which performs well on a range of document lengths (including shorter text) and determines the single most likely topic associated with each input. For each memory-collection set (total of 5 sets), we ran 500 iterations of the topic model to ensure convergence. We retained default hyperparameter values (0.1 for alpha and beta) and varied the maximum number of topics from 20 to 40 topics to determine which number yielded the highest coherence score and interpretability of topics. As our coherence measure, we used UCI-coherence which calculates point-wise mutual information and word co-occurrence within a sliding window (21), which we set to be 10 words. We implemented this procedure on each memory collection: (1) memory for 2020 (collected December 2020): max. topics = 20, UCI = -1.49; (2) memory for 2020 (+1 year later): max. topics = 30, UCI = -2.37; (3) memory for 2020 (+2 years later): max. topics = 30, UCI = -2.5; (4) memory for 2020 (+2 years later, replication set): max. topics = 30, UCI = -1.6; (5) memory for 2021 (collected December 2021): max. topics = 20, UCI = -2.9.

The first (and largest) memory collection yielded seven topics (excluding topics that explained less than 1% of the data). We therefore inferred the category of the top seven topics of each memory collection and visualized their distribution across month (Fig. S2 below). To directly compare generated topics across memory collections, see Table S2 for the top 10 words of each topic within each memory set.

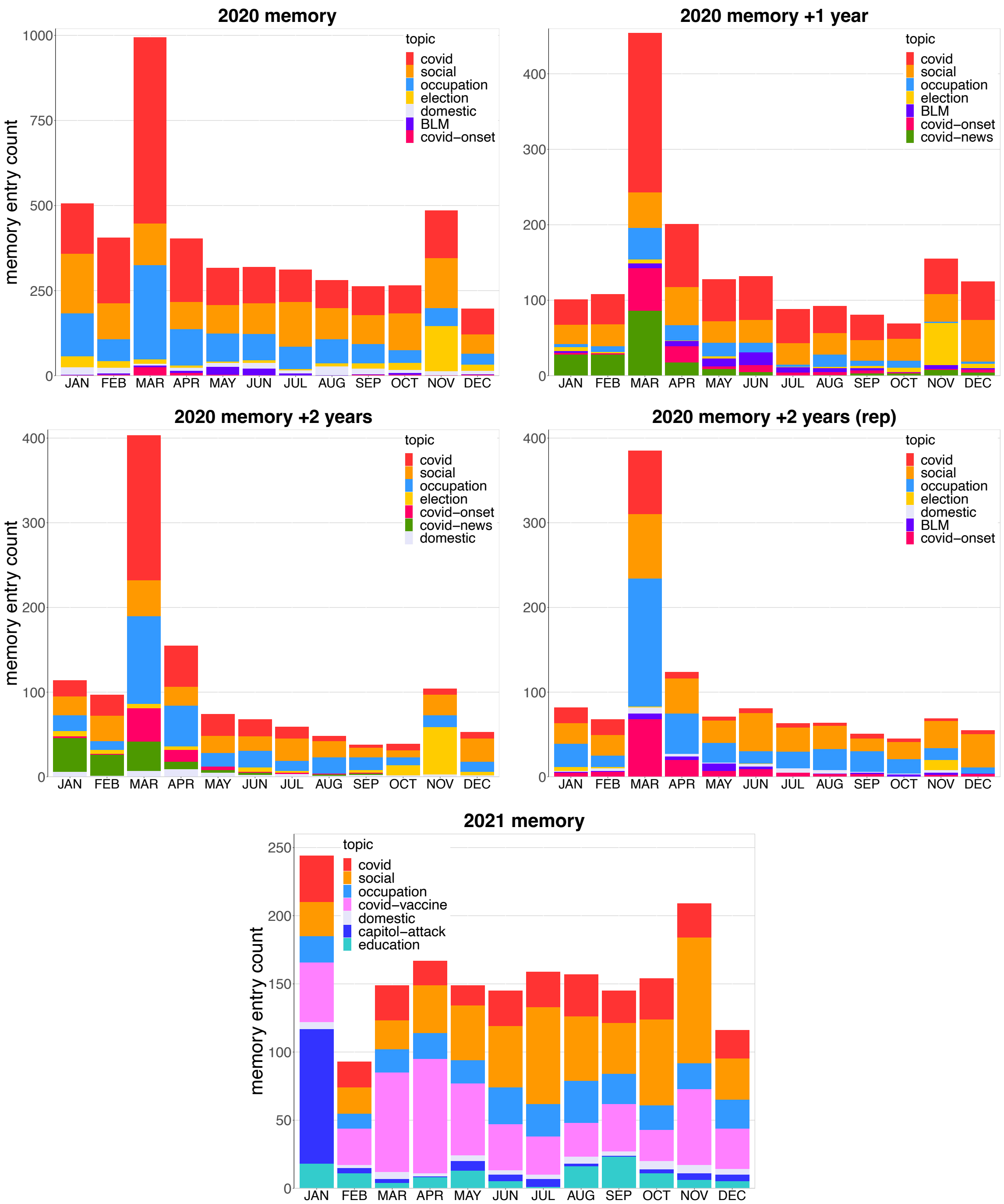

Fig. S2. Topic modeling for autobiographical memory distributed by month. Memory entry count (y-axis) represents the number of memory entries assigned to a topic category (each entry was assigned to a single predominant category). Autobiographical memory included topics relevant to personal and real-world events. More personal topics (e.g., ‘social’, ‘occupation’, ‘domestic’) were more evenly distributed across the year (with the exception of greater ‘occupation’ memories in March 2020 due to pandemic-initiated changes). Memories of real-world events (e.g. the presidential ‘election’, the January 6 ‘capitol-attack’), however, were most prevalent at the time of their occurrence. See Table S2 for keywords associated with each topic.

	2020 (longitudinal sample)	2020 +1 year (longitudinal sample)	2020 +2 years (longitudinal sample)	2020 +2 years (replication sample)	2021 (longitudinal sample)
covid	covid: 534, people: 502, work: 386, home: 385, virus: 304, march: 298, pandemic: 265, feel: 250, begin: 239, still: 231 n = 1988	covid: 348, work: 272, home: 217, people: 211, felt: 118, pandemic: 112, family: 106, friend: 95, really: 94, still: 91 n = 860	covid: 169, people: 129, home: 96, work: 77, pandemic: 71, mask: 67, store: 57, virus: 56, march: 44, grocery: 43 n = 426	covid: 117, people: 84, pandemic: 42, virus: 29, feel: 27, news: 25, world: 25, felt: 23, begin: 23, bad: 22 n = 194	covid: 53, back: 47, friend: 44, hospital: 42, die: 42, well: 40, still: 37, surgery: 35, away: 32, really: 31 n = 341
social	friend: 252, family: 234, birthday: 234, home: 220, new: 167, really: 144, celebrate: 138, visit: 134, house: 129, come: 125 n = 1327	family: 121, friend: 83, covid: 73, home: 64, daughter: 57, visit: 55, christmas: 54, together: 48, house: 48, son: 48 n = 464	family: 74, covid: 74, friend: 74, birthday: 39, home: 35, christmas: 34, able: 33, together: 31, still: 27, people: 27 n = 302	family: 112, covid: 95, friend: 82, home: 62, house: 60, back: 53, new: 41, christmas: 40, visit: 40, move: 38 n = 446	family: 149, friend: 131, visit: 107, able: 71, birthday: 63, great: 60, home: 53, trip: 49, thanksgiving: 49, travel: 47 n = 584
occupation	work: 453, school: 295, job: 203, home: 198, march: 169, back: 165, new: 145, class: 121, move: 116, online: 116 n = 1107	work: 90, school: 66, home: 53, job: 34, kid: 23, son: 21, learn: 20, pandemic: 19, march: 16, begin: 14 n = 162	work: 183, home: 144, school: 110, covid: 54, new: 52, back: 45, online: 44, house: 38, much: 38, march: 38 n = 350	work: 190, home: 156, school: 126, covid: 82, job: 73, move: 68, new: 65, pandemic: 64, online: 60, back: 56 n = 431	work: 91, job: 86, move: 69, new: 65, house: 33, home: 31, much: 27, back: 24, well: 23, really: 23 n = 285
election	election: 170, trump: 129, biden: 74, vote: 44, people: 40, president: 40, 2020: 34, presidential_election: 33, watch: 31, felt: 29 n = 308	election: 65, trump: 40, biden: 30, presidential_election: 21, vote: 15, november: 14, president: 14, win: 14, result: 13, watch: 13 n = 97	election: 65, trump: 51, biden: 43, result: 28, vote: 18, covid: 17, president: 16, watch: 13, presidential_election: 13, win: 12 n = 112	election: 19, trump: 15, biden: 13, presidential: 9, people: 6, office: 5, joe: 4, presidency: 4, vote: 4, divisive: 4 n = 24	
domestic (miscellaneous)	car: 44, home: 21, fire: 18, able: 17, back: 16, summer: 16, air: 16, come: 15, month: 15, drive: 15 n = 170		money: 27, business: 14, stock: 12, back: 12, pay: 11, pandemic: 10, still: 9, bill: 8, try: 8, covid: 8 n = 52	cat: 13, give: 10, look: 8, dog: 8, covid: 6, car: 6, end: 6, back: 5, baby: 5, much: 5 n = 42	run: 13, new: 12, back: 9, friend: 9, bought: 9, learn: 9, phone: 8, computer: 8, buy: 8, good: 6 n = 56
black lives matter (BLM)	people: 43, protest: 36, george_floyd: 27, blm: 18, police: 16, death: 14, country: 14, watch: 13, summer: 12, kill: 12 n = 87	people: 30, protest: 24, pandemic: 14, george_floyd: 14, watch: 12, blm: 12, covid: 12, happen: 11, church: 11, say: 11 n = 79		work: 26, people: 22, home: 19, police: 14, happen: 13, protest: 13, back: 12, pandemic: 10, office: 10, george_floyd: 10 n = 39	
covid-onset	toilet_paper: 21, store: 20, shelf: 14, food: 11, supply: 11, march: 10, grocery_store: 9, product: 9, item: 9, early: 9 n = 55	people: 54, store: 41, grocery_store: 29, toilet_paper: 28, food: 27, shelf: 26, pandemic: 24, use: 21, empty: 20, item: 20 n = 126	store: 41, toilet_paper: 29, find: 26, food: 21, grocery: 19, mask: 18, empty: 18, shelf: 16, supply: 16, people: 15 n = 75	people: 48, mask: 41, covid: 35, grocery_store: 31, work: 28, toilet_paper: 27, store: 26, lockdown: 26, grocery: 23, stock: 21 n = 142	
covid-news		covid: 114, virus: 52, pandemic: 51, news: 36, people: 34, march: 34, spread: 34, china: 29, feel: 27, bad: 25 n = 208	covid: 66, virus: 32, china: 29, news: 28, people: 23, pandemic: 21, lockdown: 19, january: 19, hearing: 18, begin: 18 n = 138	(included in covid)	
education	(included in occupation)	(included in occupation)	(included in occupation)	(included in occupation)	school: 74, back: 42, son: 22, able: 22, person: 21, work: 19, return: 17, college: 17, class: 16, covid: 15 n = 129
covid-vaccine					vaccine: 440, covid: 196, people: 134, shot: 102, still: 85, feel: 83, felt: 82, able: 70, back: 67, normal: 58 n = 583
capitol-attack					watch: 47, january: 42, capitol: 40, president: 33, trump: 32, biden: 32, people: 29, news: 26, 6: 19, election: 19 n = 147

Table S2. Topics and keywords across memory sets. The top seven topics of each memory set are represented with their top 10 keywords; darker colors represent higher topic count within that memory set (n represents the total topic count); numbers by keywords represent their count within the topic. Categories were selected based on the overall cluster of words; however, some frequently appearing topics like 'covid' or 'work'

did co-occur in topics that were not directly related to them. Moreover, across memory sets, the “domestic” category (which was relatively low frequency) varied in its (subcategory) interpretation; for example, the topic is related to financial matters in the ‘2020 +2 years (longitudinal sample)’ set, but domestic care in the replication set. Overall, generated topics were interpretable and comparable across memory collections.

2.3.2 Autobiographical Interview analysis tool. We used a new NLP tool that has fine-tuned a popular and efficient language model (DistilBERT, 22) by training it to classify autobiographical narratives for episodic content (23) according to the Autobiographical Interview method (24). This model predicts manual Autobiographical Interview scores with high accuracy (tested across five datasets). We provided as input to the model each participant’s individual memory entries (labeled by month), which generated a score for the number of “internal details” (episodic content about a central memory) or “external details” (semantic or non-episodic content); the sum of internal and external details equals the number of words for each recalled month. We used the raw number of internal and external details in our analysis instead of a single normalized score, as the amount of detail for each may be a more sensitive and informative measure. We note that using a non-normalized score introduced outliers, which we accounted for across our analyses.

2.3.3 Sentiment analysis tool. We analyzed the sentiment of autobiographical memory using the Valence Aware Dictionary and sEntiment Reasoner (VADER, 25), a well-established, rule-based model and lexicon trained on social media content. The model took the tokenized memories as input and generated a sentiment score, reflecting polarity (valence: positive/neutral/negative) as well as sentiment strength (i.e., the estimated proportion of positive versus neutral versus negative sentiment; sums to 1), of each sentence. In our analysis, we used the compound score, ranging from -1 (negative) to 1 (positive) to test our predictions.

2.3.4 Event segmentation model. In order to verify whether the observed temporal patterns in autobiographical memory reflected distinct events, we implemented a recently developed approach that uses GPT-3 (26) to segment events from narratives and that successfully predicts human-generated event segmentation (27). We followed their procedure (including the exact parameter settings) and fed GPT-3 (model version: ‘text- davinci-002’) the following prompt: "*An event is an ongoing coherent situation. The following story needs to be copied and segmented into events. Copy the following story word-for-word and start a new line whenever one event ends and another begins. This is the story:* ” along with autobiographical memory entries (iterating through a single memory entry at a time). This measure correlated with our human-generated segmentation of episodic events (r = 0.88; SI Appendix 3.2.3). Moreover, we tested this event-count measure (instead of the participant-generated memory entry) in our statistical models and replicated all results (SI Appendix 3.2.2).

3. Replication analysis

3.1 Replication sample. To ensure the generalizability of our findings, we recruited a new sample of participants (N = 252) to compare to our longitudinal cohort (N = 303) during our third memory collection (January 2023). These participants were naïve to the COVID-Dynamic project and our memory tasks. We first analyzed memory of the replication group separately, and then evaluated whether the two groups significantly differed in their memory patterns by testing for an interaction between group (replication/longitudinal sample) and the effects of interest.

In the replication sample, we again found a pronounced memory bump for March 2020, which was no different from that of our longitudinal sample ($\beta = 1.68$, $z = 27.61$, $p < 0.001$; M = 1.68 [1.56, 1.80]; interaction: $\beta = 0.10$, $z = 1.18$, $p = 0.24$; M = 0.10 [-0.07, 0.26]; Fig. S3A). We also found their March memories to be recalled earlier, similar to our original cohort ($\beta = -2.29$, $t = -9.89$, $p < 0.001$; M = -2.29 [-2.75, -1.81]; interaction: $\beta = -0.47$, $t = -1.59$, $p = 0.11$; M = -0.47 [-1.03, 0.11]; Fig. S3C). We further replicated the characteristics of the content for March-2020 memory. March memories included more episodic details ($\beta = 25.12$, $t = 9.59$, $p < 0.001$; M = 25.12 [20.25, 30.14]; interaction: $\beta = 3.62$, $t = 1.06$, $p = 0.29$; M = 3.53 [-3.13, 10.26]), and contained more negative sentiment ($\beta = -0.05$, $t = -2.55$, $p = 0.01$; M = -0.06 [-0.10, -0.01], interaction: $\beta = -0.01$, $t = -0.45$, $p = 0.66$, M = -0.01 [-0.07, 0.05]). Taken together, we replicated our autobiographical memory results in a new sample (retrieved two years after the end of 2020), enhancing the generalizability of these results.

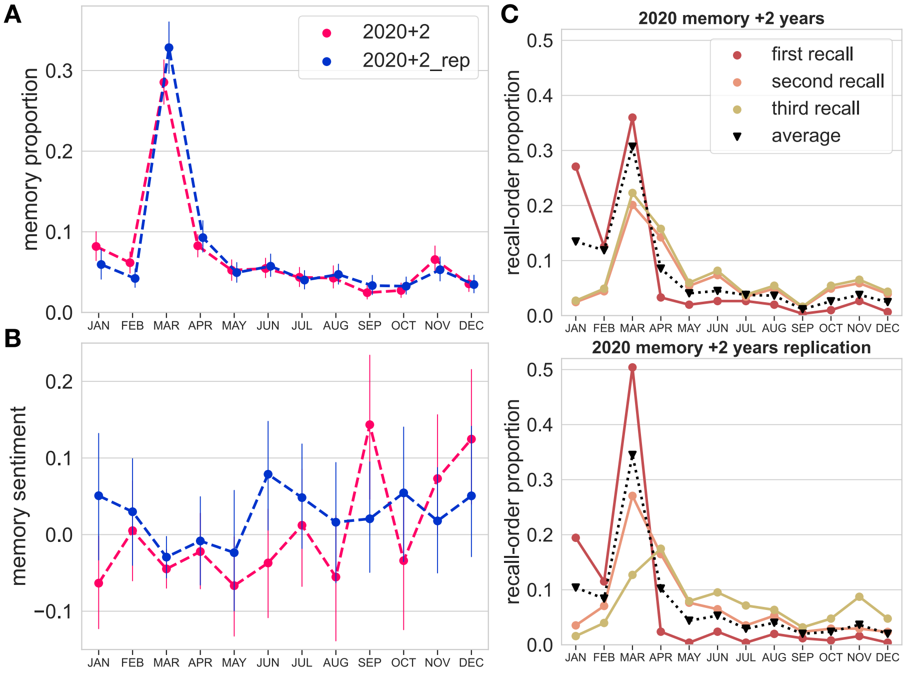

Fig. S3. Comparison between longitudinal and replication sample for 2020 memory. Two years after the end of 2020, we collected autobiographical memory in our longitudinal sample for a third time ('2020+2') and

in a new, 'replication' sample ('2020+2_rep'). We replicated our main findings: March 2020 was (A) preferentially recalled, leading to a substantial "bump" in autobiographical memory, (B) was more negative in sentiment, and (C) was recalled earlier than other months of 2020.

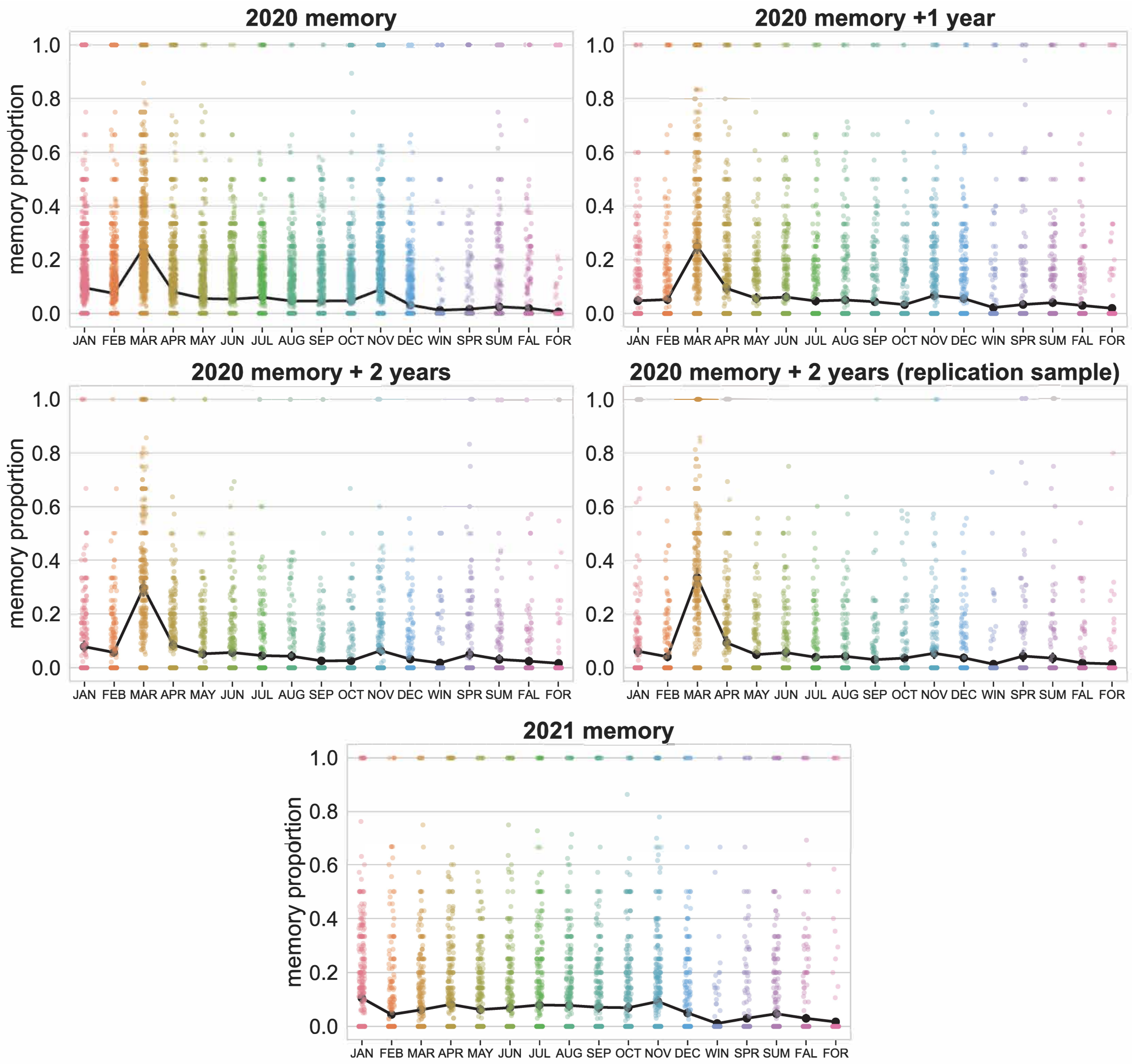

Fig. S4. Distribution of recollections across all possible date options. Individual-level data for Fig. 2A in main manuscript by memory collection. Within-participant proportion of autobiographical memory across participant-dated calendar months, 'approximate' date options ('WIN': approximately winter; 'SPR': approximately spring; 'SUM': approximately summer; 'FAL': approximately fall), and "I do not remember" date option (i.e., forgotten, 'FOR'). For a description of results, see Fig. 2A in the main manuscript.

3.2 Supporting measures

3.2.1 Episodic content. We next characterized the episodic content of recalled events by implementing an NLP model that automatically scores autobiographical memory (SI Appendix 2.3.2; 23). This method reliably extracts the 'internal' or episodic details of memory, such as specific details of the location, time, people, actions and thoughts, versus 'external' details, which

convey more general or factual statements that can be unrelated to the central memory. For our analysis, we used the subject-level internal-detail score for each recalled month. Mirroring memory-count results, we found that for recalled months, there were more estimated internal details for March versus other months of 2020, when recalled in December 2020 ($\beta = 14.12$, $t = 7.47$, $p < 0.001$; M = 14.19 [10.40, 17.83]), one year later ($\beta = 11.22$, $t = 5.60$, $p < 0.001$; M = 11.22 [7.17, 15.16]), and two years later ($\beta = 21.51$, $t = 9.71$, $p < 0.001$; M = 21.56 [17.20, 25.87]; also replicated in new sample of participants: SI Appendix 3.1).

We did not observe this pattern for 2021 memory ($\beta = -3.62$, $t = -1.57$, $p = 0.12$; M = -3.64 [-8.22, 0.89]; interaction between month and year recalled: $\beta = -18.10$, $t = -4.21$, $p < 0.001$; M = -18.01 [-26.39, -9.57]). As participants generally wrote more about March 2020, the number of external details followed the same pattern; nevertheless, we focused on internal details to verify whether these memories contained episodic content at all. These results confirmed that increased memory for March 2020 included episodic details, putatively engaging rich, detailed memory associated with the hippocampus (28).

3.2.2 GPT-3 event count measure. We tested our pattern of results using GPT-3 model-generated event count to approximate the number of distinct events described (SI Appendix 2.3.4). We replicated all results for each memory collection. Specifically, we found greater event count for March 2020 in every memory collection (*2020 memory*, $\beta = 1.18$, $z = 58.65$, $p < 0.001$; M = 1.18 [1.14, 1.22]; *2020 +1 year memory*, $\beta = 1.38$, $z = 44.83$, $p < 0.001$; M = 1.38 [1.32, 1.44]; *2020 +2 years memory*, $\beta = 1.63$, $z = 47.67$, $p < 0.001$; M = 1.63 [1.56, 1.69]; *2020 +2 years memory (replication sample)*, $\beta = 1.71$, $z = 47.14$, $p < 0.001$; M = 1.71 [1.64, 1.79]), whereas we did not find this pattern for 2021 memory ($\beta = -0.09$, $z = -1.79$, $p = 0.07$; M = -0.09 [-0.20, 0.01]).

We further replicated key interactions showing a significant difference between this effect for March 2020 versus 2021 memory ($\beta = -1.27$, $z = -22.66$, $p < 0.001$; M = -1.27 [-1.38, -1.16]), as well as for even greater (relative) March-2020 memory in the third memory collection versus the first ($\beta = 0.45$, $z = 11.25$, $p < 0.001$; M = 0.45 [0.37, 0.53]) and the second memory collection ($\beta = 0.25$, $z = 5.36$, $p < 0.001$; M = 0.25 [0.16, 0.34]). Lastly, we again did not find a difference in this effect between our longitudinal and replication sample (*2020 +2 years*: $\beta = 0.09$, $z = 1.71$, $p = 0.09$; M = 0.09 [-0.01, 0.18]).

3.2.3 Human rater verification of episodic-event count. To verify that our memory count measures approximated human episodic event segmentation, we recruited five independent raters to segment and count the number of episodic events within the autobiographical memories of 10 participants each (50 participants in total). We provided the same instructions that were given to GPT-3 in defining an event as “an ongoing coherent situation” (SI Appendix 2.3.4), and

further defined an ‘episodic’ (versus non-episodic) memory with criteria from the Autobiographical Interview (24). Raters were tasked to count the number of only episodic events. We found that human episodic event-segmentation count correlated strongly with GPT-3 event count (r = 0.88) verifying this event-count measure. Moreover, and consistent with patterns of episodic detail (3.2.1), these ratings also provided evidence that greater memory for March 2020 contained episodic events.

4. Results tables

		mixed-effects logistic regression			Bayesian mixed-effects Bernoulli regression	
**questionnaire**	**measure**	**estimate**	***z*-value**	***p*-value**	**M**	**CI**
PANAS	negative affect	0.08	2.33	0.02	0.09	[0.01, 0.16]
PANAS	positive affect	-0.05	-1.35	0.18	-0.05	[-0.13, 0.03]
PANAS	positive X negative	-0.13	-1.89	0.058	-0.13	[-0.27, 0.00]
STAI	state anxiety	0.11	2.85	0.004	0.11	[0.03, 0.18]
STAI	trait anxiety	0.15	3.32	< 0.001	0.16	[0.06, 0.26]
PSS	stress	0.16	3.98	< 0.001	0.16	[0.08, 0.24]
BDI	depression	0.10	2.19	0.03	0.10	[0.01, 0.19]
RISC	resilience	-0.06	-1.27	0.21	-0.06	[-0.16, 0.04]
(face-icon) mood measure	mood	-0.04	-1.08	0.28	-0.04	[-0.11, 0.03]

Table S3. Negative affect increased likelihood of later retrieving that month, statistical results. Each row represents output from two models (frequentist and Bayesian; see 2.1 'Statistical models' above) predicting the likelihood of retrieving memories from a month in 2020 given the average affect (specified in 'measure') that had been reported for that month (questionnaires collected from April through December 2020; see 'Monthly measures' in main manuscript). 'X' indicates interactive effects. Magenta color indicates significant effects.

		mixed-effects linear regression			Bayesian mixed-effects regression	
**questionnaire**	**measure**	**estimate**	***t*-value**	***p*-value**	**M**	**CI**
PANAS	negative affect	-0.03	-3.83	<0.001	-0.03	[-0.04, -0.01]
PANAS	positive affect	0.02	2.86	0.004	0.02	[0.01, 0.04]
PANAS	positive X negative	-0.05	-3.15	0.002	-0.04	[-0.07, -0.02]
STAI	state anxiety	-0.03	-4.61	<0.001	-0.03	[-0.05, -0.02]
STAI	trait anxiety	-0.04	-4.95	<0.001	-0.04	[-0.06, -0.03]
PSS	stress	-0.03	-4.44	<0.001	-0.03	[-0.05, -0.02]
BDI	depression	-0.04	-4.95	<0.001	-0.04	[-0.06, -0.03]
RISC	resilience	0.04	4.03	<0.001	0.04	[0.02, 0.06]
(face-icon) mood measure	mood	-0.03	-4.55	<0.001	-0.03	[-0.05, -0.02]

Table S4. Affect influenced sentiment of retrieved memory, statistical results. Each row represents output from two models (frequentist and Bayesian; see 2.1 'Statistical models' above) predicting the sentiment

(ranging from -1: negative, to 1: positive) of a remembered month in 2020 given the average affect (specified in 'measure') that had been reported for that month (questionnaires collected from April through December 2020; see 'Monthly measures' in main manuscript). 'X' indicates interactive effects. Magenta color indicates significant effects.

	Poisson regression			Bayesian Poisson regression	
**measure**	**estimate**	***z*-value**	***p*-value**	**M**	**CI**
negative low arousal	0.10	12.55	<0.001	0.10	[0.09, 0.12]
negative high arousal	0.03	3.07	0.002	0.03	[0.01, 0.04]
negative self	0.08	9.25	<0.001	0.08	[0.06, 0.09]
positive low arousal	-0.06	-6.44	<0.001	-0.06	[-0.07, -0.04]
positive high arousal	-0.06	-7.23	<0.001	-0.06	[-0.08, -0.05]
positive self	-0.05	-5.59	<0.001	-0.05	[-0.06, -0.03]

Table S5. Six factors predicted the amount of recall, statistical results. Factor analysis yielded six factors explaining variance across all affect measures (see 2.2 'Factor analysis' above) and subject-level factor scores were calculated. Each row represents output from two models (frequentist and Bayesian; see 2.1 'Statistical models' above) testing whether individual factor scores predicted the overall number of memories reported. Magenta color indicates significant effects.

	linear regression			Bayesian linear regression	
**measure**	**estimate**	***t*-value**	***p*-value**	**M**	**CI**
negative low arousal	-0.03	-4.00	<0.001	-0.03	[-0.04, -0.01]
negative high arousal	-0.01	-2.05	0.04	-0.01	[-0.03, -0.001]
negative self	-0.03	-3.86	<0.001	-0.03	[-0.04, -0.01]
positive low arousal	0.03	4.29	<0.001	0.03	[0.02, 0.04]
positive high arousal	0.02	2.87	0.004	0.02	[0.01, 0.03]
positive self	0.02	3.01	0.003	0.02	[0.01, 0.03]

Table S6. Six factors predicted the sentiment of recall, statistical results. Factor analysis yielded six factors explaining variance across all affect measures (see 2.2 'Factor analysis' above) and subject-level factor scores were calculated. Each row represents output from two models (frequentist and Bayesian; see 2.1 'Statistical models' above) testing whether individual factor scores predicted the average sentiment of autobiographical memories. Magenta color indicates significant effects.

	Poisson regression			Bayesian Poisson regression	
**measure**	**estimate**	***z*-value**	***p*-value**	**M**	**CI**
intrusion	0.11	13.59	<0.001	0.11	[0.09, 0.12]
avoidance	0.10	12.12	<0.001	0.10	[0.08, 0.11]
negative cognition	0.12	14.96	<0.001	0.12	[0.10, 0.13]
arousal	0.10	12.86	<0.001	0.10	[0.09, 0.12]

Table S7. PTSD symptoms predicted the amount of recall, statistical results. Each row represents output from two models (frequentist and Bayesian; see 2.1 'Statistical models' above) testing whether individual PTSD symptoms (DSM-5, 29) predicted the overall number of memories reported. Magenta color indicates significant effects.

	linear regression			Bayesian linear regression	
**measure**	**estimate**	***t*-value**	***p*-value**	**M**	**CI**
intrusion	-0.02	-2.40	0.02	-0.02	[-0.03, -0.001]
avoidance	-0.02	-2.55	0.01	-0.02	[-0.03, -0.001]
negative cognition	-0.02	-3.25	0.001	-0.02	[-0.03, -0.01]
arousal	-0.02	-3.15	0.002	-0.02	[-0.03, -0.01]

Table S8. PTSD predicted the sentiment of recall, statistical results. Each row represents output from two models (frequentist and Bayesian; see 2.1 'Statistical models' above) testing whether individual PTSD symptoms (DSM-5, 29) predicted the average sentiment of memories reported. Magenta color indicates significant effects.

5. Covid-Dynamic Project

5.1 Description. The Covid-Dynamic study (8) was launched in April 2020 with the goal of characterizing the psychological and behavioral changes brought on by the COVID-19 pandemic in 1000+ U.S. residents. The project has administered 18 waves of data collection, completed in December 2021 (see Fig. 1A in main manuscript for collections across 2020). During each wave, participants completed a battery of measures, including assessments of emotional state and mental health, experimental tasks (e.g., standard decision-making tasks), as well as evaluations of racial, political, social, moral, and COVID-19 related attitudes. Resources and data are available to the public.

Covid-Dynamic resources:

a) Web-based data explorer: http://coviddynamicdash.caltech.edu/shiny/coviddash/
b) Information about accessing data: https://coviddynamic.caltech.edu/data-sharing
c) Summary of COVID-19 psychological studies: https://coviddynamic.caltech.edu/resources/other-covid-studies

5.2 Exploratory analysis. We investigated whether individual differences in the impact of the pandemic predicted the strength of the memory bump for March 2020. Our measure came from “The epidemic-pandemic impacts inventory” (30), collected July 2020, which assessed the personal impact of COVID-19 within 10 different domains: (1) work and employment, (2) education and training, (3) home life, (4) social activities, (5) economic, (6) emotional health and well-being, (7) physical health problems, (8) physical distancing and quarantine, (9) infection history, and (10) positive change. We aggregated the number of “yes” responses to listed changes (occurred to “me”) within each domain. Our memory measure was the individual proportion of March memory, collected December 2020. To note, 652 out of 939 participants (subjects in first memory collection) demonstrated a higher proportion of March memory than expected from a uniform distribution across all date options (17 total date options: 12 calendar months, 4 approximate seasons, and 1 forgotten-date option; 1/17 = 0.06), demonstrating that this analysis would be likely testing the strength, but not occurrence of the memory bump. We implemented Bonferroni correction for multiple comparisons (10 tests) requiring a threshold of $p < 0.005$ to pass significance. To test proportion data, we used a generalized linear model, specifying a quasi-binomial distribution weighted by individual memory count.

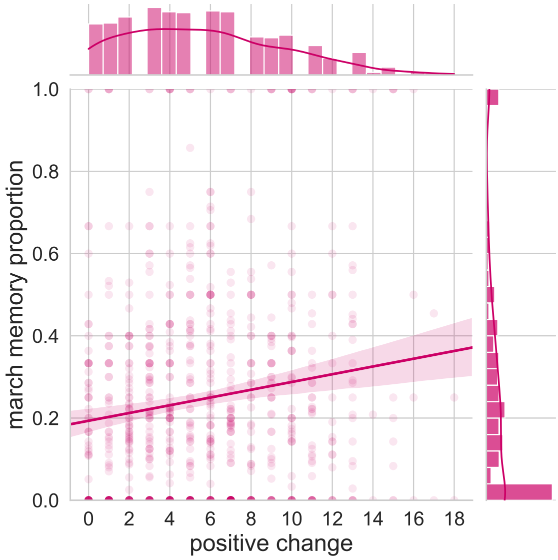

Fig. S5. Self-reported positive impact predicted further increases in March-2020 memory. Individual differences in the strength of the memory bump for March 2020 were predicted by a greater number of self-reported positive changes brought on by the COVID-19 pandemic.

We found that a greater number of reported positive changes due to the COVID-19 pandemic predicted increases in the March-memory bump ($\beta = 0.06$, $t = 5.74$, $p < 0.001$; Fig. S5 above). Moreover, this effect was present when only testing individuals already showing the memory bump ($\beta = 0.06$, $t = 5.29$, $p < 0.001$), pointing to an additive (rather than an explanatory) effect on increased memory for March 2020. This individual-difference finding is in line with reliable "reminiscence bumps" in autobiographical memory whereby individuals are more likely to remember positive transitional events in their lives (31).

5.3 Acknowledgments (extended). We thank all members of the Covid-Dynamic team who contributed to the development of the Covid-Dynamic project and acquisition of these longitudinal data, in alphabetical order: R. Michael Alvarez, Isabella Camplisson, Yanting Han, Laura Harrison, Denise Hien, Amber Hopkins, Tian Lan, Caroline Lawrence, Dehua Liang, Chujun Lin, Teresa Lopez-Castro, Uri Maoz, Marie-Christine Nizzi, Lynn K. Paul, Allison Rabkin Golden, Tessa Rusch, Damien A. Stanley, Iman Wahle, and Gideon Yaffe. See Table S9 below for specific contributions.

	Nina Rouhani	Damian Stanley	Ralph Adolphs	Uri Maoz	Lynn Paul	Tessa Rusch
Conceptualization: Initial inception of paper	3	0	2	0	0	0
Conceptualization: Composition and scope of article	3	0	2	0	0	0
Data acquisition	3	3	1	0	3	3
Data curation, Q/A, sharing	2	2	0	0	3	1
Data analysis	3	0	0	0	0	0
Funding acquisition	3	1	3	3	0	0
Methodology: Questionnaire and task selection/development	3	2	0	1	2	3
Supervision	0	0	1	0	0	0
Writing: Original draft	3	0	1	0	0	0
Writing: Review and editing	3	2	2	0	0	0
Conceptualization: Initial inception of Covid-Dynamic project	0	3	3	2	1	3
Conceptualization: Composition and scope of Covid-Dynamic project	0	3	0	2	3	3

Table S9. Author contribution table. Contribution to the present manuscript and the Covid-Dynamic project at large. Higher numbers and darker colors indicate greater contribution.

6. References

1. Google Trends. *https://trends.google.com/trends/?geo=us* (September 7, 2022).

2. A. Mavragani, G. Ochoa, K. P. Tsagarakis, Assessing the Methods, Tools, and Statistical Approaches in Google Trends Research: Systematic Review. *J. Med. Internet Res.* **20**, e9366 (2018).

3. M. Effenberger, *et al.*, Association of the COVID-19 pandemic with Internet Search Volumes: A Google TrendsTM Analysis. *Int. J. Infect. Dis.* **95**, 192–197 (2020).

4. S. Springer, L. M. Menzel, M. Zieger, Google Trends provides a tool to monitor population concerns and information needs during COVID-19 pandemic. *Brain Behav. Immun.* **87**, 109–110 (2020).

5. D. Bates, M. Mächler, B. Bolker, S. Walker, Fitting Linear Mixed-Effects Models Using lme4. *Journal of Statistical Software, Articles* **67**, 1–48 (2015).

6. P.-C. Bürkner, brms: An R package for Bayesian multilevel models using Stan. *J. Stat. Softw.* **80**, 1–28 (2017).

7. B. Ripley, *et al.*, Package 'mass.' *Cran r* **538**, 113–120 (2013).

8. T. Rusch, *et al.*, COVID-Dynamic: A large-scale multifaceted longitudinal study of socioemotional and behavioral change across the pandemic. *Scientific Data* (2022, in press).

9. D. Watson, H. Levin-Aspenson, Positive and Negative Affect Schedule (PANAS). *Encyclopedia of Clinical Neuropsychology*, 1–3 (2018).

10. C. D. Spielberger, R. L. Gorsuch, *Manual for the state-trait anxiety inventory (form Y):(" self-evaluation questionnaire")* (Consulting Psychologists Press, Incorporated, 1983).

11. A. T. Beck, R. A. Steer, G. K. Brown, *BDI-II, Beck Depression Inventory: Manual* (Psychological Corporation, 1996).

12. S. Cohen, T. Kamarck, R. Mermelstein, A global measure of perceived stress. *J. Health Soc. Behav.* **24**, 385–396 (1983).

13. K. M. Connor, J. R. T. Davidson, Development of a new resilience scale: the Connor-Davidson Resilience Scale (CD-RISC). *Depress. Anxiety* **18**, 76–82 (2003).

14. E. M. Buchanan, K. D. Valentine, S. E. Schulenberg, *Exploratory and confirmatory factor analysis: Developing the purpose in life test--short form* (SAGE Publications, Ltd., 2014).

15. S. Tobias, J. E. Carlson, Brief report: Bartlett's test of sphericity and chance findings in factor analysis. *Multivariate Behav. Res.* **4**, 375–377 (1969).

16. G. Raiche, D. Magis, nFactors: An R package for parallel analysis and non graphical solutions to the Cattell scree test. *R package version*.

17. W. Revelle, Others, psych: Procedures for psychological, psychometric, and personality research. *R package version* **1** (2018).

18. Y. Rosseel, lavaan: An R Package for Structural Equation Modeling. *J. Stat. Softw.* **48**, 1–36 (2012).

19. S. Bird, E. Klein, E. Loper, *Natural Language Processing with Python: Analyzing Text with the Natural Language Toolkit* ("O'Reilly Media, Inc.," 2009).

20. J. Yin, J. Wang, A dirichlet multinomial mixture model-based approach for short text clustering in *Proceedings of the 20th ACM SIGKDD International Conference on Knowledge Discovery and Data Mining*, KDD ’14., (Association for Computing Machinery, 2014), pp. 233–242.

21. D. Newman, Y. Noh, E. Talley, S. Karimi, T. Baldwin, Evaluating topic models for digital libraries. *Proceedings of the 10th annual joint conference on Digital libraries* (2010) https:/doi.org/10.1145/1816123.1816156.

22. V. Sanh, L. Debut, J. Chaumond, T. Wolf, DistilBERT, a distilled version of BERT: smaller, faster, cheaper and lighter. *arXiv [cs.CL]* (2019).

23. R. van Genugten, D. L. Schacter, Automated Scoring of the Autobiographical Interview with Natural Language Processing (2022) https:/doi.org/10.31234/osf.io/nyurm.

24. B. Levine, E. Svoboda, J. F. Hay, G. Winocur, M. Moscovitch, Aging and autobiographical memory: dissociating episodic from semantic retrieval. *Psychol. Aging* **17**, 677–689 (2002).

25. C. Hutto, E. Gilbert, VADER: A Parsimonious Rule-Based Model for Sentiment Analysis of Social Media Text. *ICWSM* **8**, 216–225 (2014).

26. T. B. Brown, *et al.*, Language Models are Few-Shot Learners. *arXiv [cs.CL]*, 1877–1901 (2020).

27. S. Michelmann, M. Kumar, K. A. Norman, M. Toneva, Large language models can segment narrative events similarly to humans. *arXiv [cs.CL]* (2023).

28. L. Davachi, Item, context and relational episodic encoding in humans. *Curr. Opin. Neurobiol.* **16**, 693–700 (2006).

29. J. Sveen, K. Bondjers, M. Willebrand, Psychometric properties of the PTSD Checklist for DSM-5: a pilot study. *Eur. J. Psychotraumatol.* **7**, 30165 (2016).

30. D. J. Grasso, M. J. Briggs-Gowan, J. D. Ford, A. S. Carter, The epidemic--pandemic impacts inventory (EPII). *Conn. Dent. Stud. J.* (2020).

31. D. Berntsen, D. C. Rubin, Emotionally charged autobiographical memories across the life span: the recall of happy, sad, traumatic, and involuntary memories. *Psychol. Aging* **17**, 636–652 (2002).
