# Supplementary material for: Collective events and individual affect shape autobiographical memory
Source: Proc Natl Acad Sci U S A. 2023 Jul 11;120(29):e2221919120. doi: 10.1073/pnas.2221919120 (PMC10629560; doi:10.1073/pnas.2221919120)
Supplement: Supplementary file 2 — Dataset S01 (DOCX) [file pnas.2221919120.sd01.docx]

COVID-Dynamic Team (list of authors and contributions in SI Appendix Table S9)

**Uri Maoz**

Division of Biology and Biological Engineering, California Institute of Technology, Pasadena, CA, USA

Institute for Interdisciplinary Brain and Behavioral Sciences, Crean College of Health and Behavioral Sciences, Schmid College of Science and Technology, Chapman University, Orange, CA, USA

**Lynn Paul**

Division of Humanities and Social Sciences, California Institute of Technology, Pasadena, CA, USA

**Tessa Rusch**

Division of Humanities and Social Sciences, California Institute of Technology, Pasadena, CA, USA
